# Physico-chemical properties of manufactured nanomaterials - Characterisation and relevant methods. An outlook based on the OECD Testing Programme

**DOI:** 10.1016/j.yrtph.2017.10.019

**Published:** 2018-02

**Authors:** Kirsten Rasmussen, Hubert Rauscher, Agnieszka Mech, Juan Riego Sintes, Douglas Gilliland, Mar González, Peter Kearns, Kenneth Moss, Maaike Visser, Monique Groenewold, Eric A.J. Bleeker

**Affiliations:** a European Commission, Joint Research Centre, Ispra, Via E. Fermi 2749, 21027 Ispra, VA, Italy; b Organisation for Economic Co-operation and Development (OECD), Environment Directorate, 75775 Paris CEDEX 16, France; c United States Environmental Protection Agency (US-EPA), Office of Pollution Prevention and Toxics (7405M), 1200 Pennsylvania Avenue, NW, Washington DC, 20460 United States; d National Institute for Public Health and the Environment (RIVM), PO Box 1, 3720 BA Bilthoven, The Netherlands

**Keywords:** Manufactured nanomaterials, Physico-chemical properties, Characterisation, OECD test guidelines, Working party on manufactured nanomaterials, 2D, two dimensional, **3D**, three dimensional, **AES**, Auger electron spectroscopy, **AFM**, Atomic force microscopy, **ATOF-MS**, Aerosol time-of-flight mass spectrometry, **BET**, Brunauer-Emmett-Teller method. **BJH**, Barrett-Joyner-Halenda method, **CEN**, European Committee for Standardisation, **CLS**, Centrifugal liquid sedimentation, **CNT**, Carbon nanotube, **DLS**, Dynamic light scattering, **DOSY NMR**, Diffusion ordered spectroscopy nuclear magnetic resonance. **DSC**, differential scanning calorimetry, **DTA**, Differential thermal analysis, **EDX (EDS)**, Energy Dispersive X-ray spectrometry, **ELS**, Electrophoretic light scattering, **EM**, electron microscopy, **EPR**, electron paramagnetic resonance, **ESR**, electron spin resonance, **FFF**, Field flow fractionation, **FTIR**, Fourier-transform infrared spectroscopy, **GD**, guidance document, **ICP**, Inductively coupled plasma, **ISO**, International Organisation for Standardisation, **JRC**, European Commission's Joint Research Centre, **MALS**, Multi-angle light scattering, **MS**, mass spectrometry, **MWCNT**, multi-walled carbon nanotube, **NM**, nanomaterial, **NMR**, Nuclear magnetic resonance, **OECD**, Organisation for Economic Co-operation and Development, **OES**, Optical emission spectrophotometry, **ORP**, oxidation, reduction potential, **POM**, Polarised Light Optical Microscopy, **ROS**, reactive oxygen species, **SAXS**, Small angle X-ray scattering, **SD**, small drum method, **SDR**, Sensor disk reader, **SEM**, Scanning electron microscopy, **SIMS**, Secondary ion mass spectrometry, **SMPS**, Scanning mobility particle spectrometry, **SP**, Single particle, **SSA**, specific surface area, **STM**, Scanning tunnelling microscopy, **SWCNT**, single-walled carbon nanotube, **TEM**, transmission electron microscopy, **TG**, OECD Test Guideline, **TGP**, OECD Test Guidelines Programme, **ToF-SIMS**, Time-of-flight Secondary Ion Mass Spectrometry, **UV-vis**, Ultra violet-visible, **(U)SAXS**, (Ultra)Small Angle X-ray Scattering, **VS**, Vortex shaker method, **WAXS**, Wide angle X-ray scattering, **WPMN**, OECD Working Party on Manufactured Nanomaterials, **XPS**, X-ray photoelectron spectroscopy, **XRD**, X-ray diffraction, **XRF**, X-ray fluorescence

## Abstract

Identifying and characterising nanomaterials require additional information on physico-chemical properties and test methods, compared to chemicals in general. Furthermore, regulatory decisions for chemicals are usually based upon certain toxicological properties, and these effects may not be equivalent to those for nanomaterials. However, regulatory agencies lack an authoritative decision framework for nanomaterials that links the relevance of certain physico-chemical endpoints to toxicological effects. This paper investigates various physico-chemical endpoints and available test methods that could be used to produce such a decision framework for nanomaterials. It presents an overview of regulatory relevance and methods used for testing fifteen proposed physico-chemical properties of eleven nanomaterials in the OECD Working Party on Manufactured Nanomaterials' Testing Programme, complemented with methods from literature, and assesses the methods' adequacy and applications limits. Most endpoints are of regulatory relevance, though the specific parameters depend on the nanomaterial and type of assessment. Size (distribution) is the common characteristic of all nanomaterials and is decisive information for classifying a material as a nanomaterial. Shape is an important particle descriptor. The octanol-water partitioning coefficient is undefined for particulate nanomaterials. Methods, including sample preparation, need to be further standardised, and some new methods are needed. The current work of OECD's Test Guidelines Programme regarding physico-chemical properties is highlighted.

## Highlights

• Discussion of methods used for generating the OECD WPMN data on physico-chemical properties.
• Indication of relevance of each physico-chemical endpoint and availability of adequate methods to investigate the endpoints.
• Identification of areas for revision/new development of OECD Test Guidelines or Guidance Documents.

## 1 Introduction

In general, it is recognised that identifying and characterising nanomaterials, also for toxicological testing, requires the use of additional physico-chemical properties compared to chemicals in general. Regulatory decisions are usually based upon certain toxicological properties of non-nanoscale materials, and these effects may not be equivalent to those for nanomaterials. This paper investigates physico-chemical endpoints and available test methods that could be used to produce an authoritative decision framework which links the relevance of certain physico-chemical endpoints to toxicological effects nanomaterials. Regulatory agencies currently lack such a framework for nanomaterials.

Since 2006, the Organisation for Economic Co-operation and Development (OECD) has co-ordinated, via its Working Party on Manufactured Nanomaterials (WPMN), an extensive programme (Testing Programme) on the testing of manufactured nanomaterials. One planned outcome of the Testing Programme was information for evaluating the need for OECD Test Guidelines (TGs) addressing additional physico-chemical endpoints for nanomaterials. This programme included the testing of 11 types of nanomaterials, some covering several nanoforms, for 59 endpoints using OECD TGs and other methods. The OECD TGs are developed for regulatory purposes and are agreed upon under the OECD Mutual Acceptance of Data (OECD, 1981a), which is a legally binding instrument to facilitate the international acceptance of information for the regulatory safety assessment of chemicals. A main aim of the Testing Programme was to explore the usefulness of OECD TGs and assess the need for updated or new OECD TGs. The materials tested were Au, Ag (both colloidal solutions), TiO_2_, SiO_2_, CeO_2_, ZnO, fullerenes (C_60_), SWCNTs, MWCNTs, nanoclays (all in powder form) and dendrimers (liquid). The endpoints covered addressed physico-chemical properties, environmental fate, and human and environmental hazard properties. The outcomes of the testing are presented in publicly available dossiers that contain an overview of all the materials tested and the raw data for each type of material (OECD, 2016e). Rasmussen et al. (2016) give an overview of the achievements of the WPMN Testing Programme, including the physico-chemical testing. Early in its work the WPMN published a first preliminary evaluation of the applicability of the OECD TGs to nanomaterials (OECD, 2009), which concluded that a significant number of the TGs for testing physico-chemical properties of general chemicals are not applicable to nanomaterials.

To evaluate the relevance of the proposed additional physico-chemical endpoints and the method(s) applied for testing these, the OECD organised two expert meetings, one in 2013 in collaboration with ISO/TC 229 (Nanotechnologies) (OECD, 2014a) to address, from a regulatory perspective, specific issues relevant to physico-chemical properties of manufactured nanomaterials, and a second one in 2014 to address the identification of appropriate methods for physico-chemical parameters (OECD, 2016b). Where possible, the second meeting identified appropriate test methods for both a particular parameter and particular types of nanomaterials (OECD, 2016b). Furthermore, a group of experts under the WPMN evaluated the methods used in the Testing Programme to identify the most promising methods for harmonisation within the OECD (OECD, 2016a). Also the WPMN expert meeting on environmental issues (OECD, 2014b) discussed the physico-chemical properties.

Recognising the importance of testing the same material for all endpoints to minimise uncertainties regarding differences between samples tested by different laboratories, as well as potential issues concerning sampling and homogenisation, the European Commission's Joint Research Centre (JRC) established the JRC Nanomaterials Repository (Totaro et al., 2016), which supplied materials to the Testing Programme. A comprehensive data set on physico-chemical characterisation of these nanomaterials was also developed (Singh et al., 2011, Rasmussen et al., 2013, Rasmussen et al., 2014a, Rasmussen et al., 2014b, Singh et al., 2014).

In addition to the OECD WPMN work, national and regional initiatives (e.g. Christensen and Larsen, 2013), including projects under the EU 7th framework programme and Horizon 2020, have produced evaluations and information on suitable methods and a vast amount of data on nanomaterials.

In parallel with the WPMN work and building also on other research, consensus appears to be reached in the scientific community in recent years that the key properties for characterising nanomaterials can be organised in three main groups of properties (Stone et al., 2014, Oomen et al., 2015, ISO, 2012a):

- Characterisation (“what they are”): both physical and chemical identification in terms of composition, impurities, size and size distribution, shape, surface characteristics (coating, chemistry, functionalization, surface charge), surface area, porosity, etc.
- Fate (“where they go”): biological (toxicokinetics, bio-distribution) and environmental fate described by solubility (water solubility and rate of dissolution in relevant media), hydrophobicity, dispersibility, dustiness, etc.
- (Re)activity (“what they do”): their reactivity, physical hazards, biological reactivity, toxico-dynamics, photo-reactivity, etc.

These three classes of properties aim to support safety assessment of nanomaterials for which relevant information includes parameters for characterisation, fate (environmental fate and toxicokinetics) and inherent (re)activity in addition to (eco)toxicological data. In principle, each form of a nanomaterial has to be characterised and then tested for all relevant (eco)toxicological endpoints to obtain reliable safety assessments, which may require additional characterisation in the test systems themselves. However, in view of the potential huge number of different nanomaterials and nanoforms such an approach is probably not feasible. Therefore, a considerable effort is invested in the development of approaches for categorisation, grouping and read-across of nanomaterials based on physico-chemical properties (e.g. Arts et al., 2015, ECHA et al., 2016, Gebel et al., 2014, OECD, 2016c, OECD, 2016d, Oomen et al., 2015, Sellers et al., 2015, Walser and Studer, 2015).

The small size and relatively large specific surface area of nanomaterials may lead to differences in kinetics and magnitude of any effects when comparing a nanomaterial with its corresponding non-nanoform (if existing). This may also be the case for different nanoforms of the same chemical composition, in particular when the nanomaterial changes as a result of interaction(s) with the different environments it encounters during its life cycle. Among others, sufficient information and data concerning the physico-chemical characterisation of the nanomaterials is needed to enable modelling of these kinetic processes and subsequent toxicity (and to minimise testing). Therefore, the OECD Testing Programme included a whole range of different physico-chemical parameters to characterise the different NMs in the Programme.

Depending on the particular nanomaterial investigated, for risk assessment purposes, grouping, read-across and/or modelling additional parameters may need to be determined and taken into account as well.

The aim of this paper is to advance the understanding and indicate future needs for testing of nanomaterials with regard to physico-chemical properties based on an analysis of the methods applied in the Testing Programme and in research. Specifically, the paper:

a) Identifies which physico-chemical parameters are important for nanomaterials for regulatory purposes and which methods are available to measure them, and to indicate where certain parameters lack methods. An overview of methods applied in the Testing Programme to determine physico-chemical properties is provided, supplemented with an evaluation of whether these methods are generally applicable or whether they are material-specific. The methods are evaluated for their relevance, reliability and status of validation and standardisation.
b) Evaluates which OECD TGs and other existing standardised methods to determine physico-chemical properties should be updated, and which (if any) new physico-chemical TGs or other standardised methods should be developed.

To address this, the paper provides an overview of the physico-chemical properties proposed by the WPMN for characterisation of nanomaterials (OECD, 2010) and of methods that may be used to determine these properties (OECD, 2014a). The principles for these different methods used for testing are outlined in the Supplementary Information. The overview is primarily based on the outcomes of evaluations by the OECD (OECD, 2016a, OECD, 2016b); some methods applied to one nanomaterial only may not always be described here. Where relevant, additional methods not applied within the Testing Programme are described as well. However an extensive literature review is not attempted and thus the overview of additional methods is not exhaustive. The information is presented in three sections: characterisation of the nanomaterial, characterisation for fate assessment (environmental fate and toxicokinetics) and characterisation of inherent (re)activity, in line with the above mentioned scientific consensus on information elements that are used in grouping and read-across approaches for nanomaterials (e.g. Oomen et al., 2015, ECHA et al., 2016).

In the descriptions below, where no reference appears in conjunction with the material the information is found in the dossiers published by the OECD at http://www.oecd.org/chemicalsafety/nanosafety/dossiers-and-endpoints-testing-programme-manufactured-nanomaterials.htm. For the methods applied in the WPMN Testing Programme supplementary information was developed; for methods not applied in the Testing programme literature references are given in the text.

## 2 Characterisation of nanomaterials – physico-chemical properties

Adequate and unequivocal identification of the assessed material is the first step in any risk assessment. For non-nanomaterials, in many cases, information on chemical identity and molecular structure is sufficient to unequivocally identify the material, whereas for nanomaterials physical and other chemical characterisation appears necessary as well. If the focus is on “nano” the first step would be to determine the size and size distribution. Various definitions of “nanomaterial” and criteria to identify nanomaterials exist (see e.g. Lövestam et al., 2010, NICNAS, 2009, Health Canada, 2011, EC, 2011, US-EPA, 2015, ISO, 2015a, US-EPA, 2017), but they all agree on particle size and size distribution as essential parameters for identification, and generally the relevant size range is considered to be between 1 nm and 100 nm. The specific surface area can also aid in identifying nanomaterials (Wohlleben et al., 2017). A nanomaterial can be further characterised by its surface chemistry (i.e. the chemical nature of its surface) both in terms of composition and of functional groups, shape, crystalline phase and crystallite size and porosity of the particles. Nanoparticles resulting from application of nanotechnology that combines different layers of different chemistries into one particle, also called core-shell particles, pose additional challenges, but those are not addressed here.

## 3 Chemical characterisation

### 3.1 Chemical composition

As for non-nanomaterials, the chemical composition is a fundamental descriptor for nanomaterials, and reporting it is a regulatory requirement in all jurisdictions across the OECD. In a preliminary review of the applicability of OECD TGs for determining chemical composition to nanomaterials (OECD, 2009), only TG 101, “UV-VIS Absorption Spectra (Spectrophotometric Method)” was considered to be applicable. The WPMN concluded that the method is applicable to solutions but it is not known how the results might be affected by the fact that for nanomaterials a colloidal suspension instead of a solution may be analysed. Further work is required to elucidate this and, if necessary, to modify the TG for nanomaterials. Methods for determining the chemical composition should be carefully selected taking into consideration the (expected) composition of the NM and impurities, as some techniques are relevant only for some types of materials, e.g. organic or inorganic materials.

In the Testing Programme the following methods were applied, see also Table 1:

**Table 1:** Physico-chemical parameters for characterisation of nanomaterials and suitability of associated analytical methods.

Methods a	Available standards	Tested in OECD WPMN b	Suitable for testing nanomaterials?	Remarks
**Chemical composition** As for non-nanomaterials, the chemical composition is a fundamental descriptor for nanomaterials and it is a regulatory requirement in all jurisdictions across the OECD.				
EDX (EDS)	–	TiO_2_, SiO_2_	Yes, with restrictions	May not be suitable for nanoparticles of complex composition and in complex matrices.
ICP-OES (ICP-AES)	–	TiO_2,_ CeO_2_, ZnO, SiO_2_	Yes	Provides quantitative information. Analysis of complex samples may be difficult.
ICP-MS	CEN/TC 352 (under development); ISO/TC 229 (under development)	SiO_2_	Yes	Poor performance on lighter elements (H, I, O, N, C, Cl, Br, S).
XPS	–	–	Yes, with restrictions	(Near) surface layer must be representative for core material.
ToF-SIMS	–	CeO_2_, ZnO	Yes, with restrictions	Sample mounting (on a carbon tape) limits analysis of C and Si in samples. By principle ToF-SIMS is not quantitative, and it is rather useful to identify traces, but not to quantify them, nor to compare samples.
Other combinations of separation techniques with mass spectrometry (e.g. ToF-MS, AToF-MS, FFF)	–	–	Not evaluated	
XRD	–	–	Yes, with restrictions	XRD provides information on the crystal structure of the material investigated. The method can confirm composition, but is not suitable as a stand-alone method.
UV-VIS	–	–	Not evaluated	
FTIR, Raman, Combustion analysis	–	–	Yes, with restrictions	Are commonly used to characterise carbon-based NMs or organic NMs. FTIR may also be used for certain inorganic NMs.
**Surface chemistry** Nanomaterials can have a much larger volume specific surface area (VSSA) than bulk materials, therefore any interaction of the surface with its surroundings would be stronger for nanomaterials, including a modified activity due to a specific chemical composition of the NM surface.				
XPS	–	SWCNT, MWCNT, CeO_2_, ZnO, SiO_2_	Yes, with restrictions	Applicable to materials that are stable in ultrahigh vacuum conditions; measures composition of 0–10 nm surface layer.
EDX (EDS), SEM-EDX	–	Nanoclay TiO_2_	Yes, with restrictions	Difficult to separate properties of particle core and surface.
ToF-SIMS	–	CeO_2_, ZnO	Yes, with restrictions	Difficult to separate properties of particle core and surface; use of carbon tape for sample mounting limits the analysis of C and Si in the sample.
RBA	–	TiO_2_	Not evaluated	
FTIR	–	–	Not evaluated	Applicable also to materials that are not stable in ultrahigh vacuum conditions.
AES	–	–	Not evaluated	Applicable to materials that are stable in ultrahigh vacuum conditions; measures composition of 2–20 nm surface layer.
EELS	–	–	Not evaluated	
SIMS	–	–	Not evaluated	SIMS is considered a very sensitive qualitative technique to measure the composition of solid surfaces to a depth of 1–2 nm.
XRF/TRXF	ISO/TS 18507:2015	–	Not evaluated	Compared to XRF the TRXF method can reach detection limits in the low ppb levels or even below 1 ppb for some elements.
**Particle size distribution** Important for defining nanomaterials in regulatory context, e.g. to define whether the material fulfils the EU-definition of a nanomaterial.				
DLS	ISO 22412:2017	C_60_, SWCNT, MWCNT, Ag, Au, TiO_2,_ CeO_2_, ZnO, SiO_2,_ Dendrimers	Yes, with restrictions	Only reliable for stable particle suspensions of monomodal and relatively narrow size distributions. Results are influenced by particle shape and dispersibility. Cannot distinguish between individual particles and aggregates/agglomerates. In mixtures and polydisperse samples DLS can greatly underestimate the quantity of smaller particles.
CLS	ISO 13318-1:2001; ISO 13318-3:2004 ISO 13318-2:2007	CeO_2_, ZnO, SiO_2,_ Nanoclay	Yes, with restrictions	Results are influenced by particle shape and dispersibility. Cannot distinguish between individual particles and aggregates/agglomerates.
SEM	CEN/TC 352 (under development)	SWCNT, MWCNT, CeO_2_, ZnO	Yes, with restrictions	Applicable to materials that are stable in high-vacuum conditions. May not work well at environmentally relevant concentrations. Provides 2D-projections of 3D-particles, thus less suitable for non-spherical particles (e.g. platelets). Risk of agglomeration on the grid. Less sensitive than TEM, but easier sample preparation.
TEM	CEN/TC 352 (under development); ISO/TC 229 (under development)	C_60_, MWCNT, Ag, Au, TiO_2,_ ZnO, SiO_2,_	Yes, with restrictions	Applicable to materials that are stable in high-vacuum conditions. May not work well at environmentally relevant concentrations. Provides 2D-projections of 3D-particles, thus less suitable for non-spherical particles (e.g. platelets). Risk of agglomeration on the grid.
Laser diffraction	–	MWCNT	Yes, with restrictions	Suitable for particles >50 nm.
UV-Vis	–	Ag	Yes	This depends on the materials. UV-Vis spectroscopy would not give meaningful size results for plasmonic materials (Ag and Au). Only if the particles are monodispersed and perfectly spherical may UV-Vis spectroscopy and peak position indicate particle size for plasmonic materials, but the information cannot be considered sufficiently reliable to be used for particle size measurement.
DOSY-NMR	–	Dendrimers	Yes, with restrictions	Difficult to perform for larger particles. Not suitable for paramagnetic particles and those that would result in complex field modifications.
AFM	–	Ag, SiO_2_	Yes	Gives 3D information.
STM	–	–	Yes	Gives 3D information.
MALS	–	–	Yes	Commonly used for characterisation of proteins and polymers.
SAXS	–	TiO_2_, SiO_2_	Yes	SAXS cannot be used as a stand-alone technique for size determination, but works well for confirming results from other techniques.
Separation techniques (e.g. FFF, CHDF)	–	–	Yes	Can be used in combination with detection techniques to analyse particle size distributions.
**Specific surface area** Nanomaterials have a much larger volume specific surface area than non-nanomaterials, resulting in stronger interactions with their environment. SSA is also important in regulatory context.				
BET	ISO 9277:2010	C_60_, SWCNT, MWCNT, Ag, Au, TiO_2,_ CeO_2_, ZnO, SiO_2,_ Nanoclay	Yes, with restrictions	Applicable to dry solid samples only. .
SAXS	ISO 17867:2015	TiO_2,_ SiO_2,_	No	
**Porosity** Porosity is related to the inner surface of a material, and thus relates to the surface area of nanomaterials.				
BET	ISO 15901-2:2006	TiO_2,_ CeO_2_, ZnO, SiO_2,_	Yes, with restrictions	ISO 15901-2:2006 describes the calculation of mesopore size distribution between 2 nm and 50 nm, and of macropore distribution up to 100 nm.
BJH	ISO 15901-2:2006	CeO_2_, ZnO	Yes, with restrictions	BJH is not appropriate for microporous materials.
Mercury porosimetry	ISO 15901-3:2007	C_60_, SWCNT, MWCNT	Yes, with restrictions	Not suitable for metal-containing nanomaterials that would amalgamate with mercury. The minimum equivalent pore diameter is stated to be approximately 4 nm. The maximum diameter is limited for samples having a significant depth due to the difference in hydrostatic head of mercury from the top to the bottom of the sample. For the most purposes, this limit can be regarded as 400 μm. The measurements cover inter-particle and intra-particle porosity.
SAXS	ISO 17867:2015	–	Not evaluated	
**Crystalline phase and crystallite size** Crystalline phase and crystallite size influence reactivity and toxicity of nanomaterials				
XRD	–	Ag, Au, TiO_2,_ CeO_2_, ZnO, SiO_2,_ Dendrimers	Yes	Generally accepted method to calculate average crystallite sizes (not necessarily the particle sizes) via the Scherrer equation for sizes below 100–200 nm. XRD can confirm that materials are amorphous; no size determination is possible for amorphous materials.
SAXS	ISO 17867:2015	TiO_2,_ SiO_2_	Yes	Commonly used to confirm that a material is amorphous or non-homogeneous.
TEM	–	MWCNT, C_60_, Ag, Au	No	The more polydisperse the material, the more beam time is needed.
DSC	–	Dendrimers	No	Does not give information on crystalline phase or crystallite size. Provides complementary information on crystal phase transitions.
Raman spectroscopy	–	SWCNT, MWCNT	Not evaluated	For SWCNT and MWCNT the method can provide information about bonding structure and amount of non-amorphous material.
WAXS	–	–	Not evaluated	WAXS is used for analysing highly ordered or crystalline structures.
**Shape** Shape is one of the key parameters to define a “nanoform”. Knowledge on the actual shape is also relevant in the interpretation of analytical results of methods that are based on the assumption of spherical particles.				
TEM/SEM	Image analysis by ISO 9276-6:2008	SiO_2_	Yes	Provides 2D-projections of 3D-particles. de Temmerman et al., 2012 proposed a method for measuring shape of nanoparticles
AFM	–	–	Not evaluated	Provides 2.5D-projections of 3D-particles; prone to errors due to tip effects.
STM	–	–	Not evaluated	Provides 2.5D-projections of 3D-particles; prone to errors due to tip effects.

a For abbreviations and technical details of methods see Supplementary Information.

b Analytical methods have been evaluated for measurement of physico-chemical properties of specific nanomaterials within the OECD WPMN Testing Programme of Manufactured Nanomaterials as indicated.

#### 3.1.1 **ICP** (inductively coupled plasma) combined with the detection method **OES** (optical emission spectrophotometry, also referred to as inductively coupled plasma atomic emission spectroscopy, i.e. ICP-AES), or **MS** (mass spectrometry)

ICP combined with suitable detection methods is one of the best suited techniques for detailed elemental analysis of inorganic materials, including nanomaterials (Tantra, 2016). ICP-OES was applied to CeO_2_ and TiO_2_ in the Testing Programme and ICP-OES and ICP-MS were applied to nanostructured SiO_2_ (see also Rasmussen et al., 2013). ICP-OES provides quantitative information as the intensity of the optical emission is indicative of the concentration of an element within the sample (Tantra, 2016) and up to 70 elements can be analysed simultaneously. It is a generally accepted method for the detection of trace metals. ICP-OES can be used to characterise both organic and inorganic nanomaterials; however emission lines of different elements may interfere with each other, thus the analysis of complex samples may be difficult. ICP-MS and ICP-OES do not provide information on the crystal structure of the investigated material and, furthermore, the performance of ICP-MS for some of the lighter elements, such as H, I, O, N, C, Cl, Br and S, is poor due to the contributions of the background atmosphere in the flame-based measurement. ICP-MS is at least an order of magnitude more sensitive than ICP-OES; the relative performance for light elements and elements with strong interferences (e.g. Si) should be carefully checked.

#### 3.1.2 **EDX (or EDS)** (energy dispersive X-ray spectroscopy)

EDX (in both TEM and SEM) was applied to TiO_2_ in the Testing Programme (TEM: Rasmussen et al., 2014a; SEM: the dossier), and EDX with TEM was applied to nanostructured SiO_2_ (Rasmussen et al., 2013). EDX is in principle suitable for detecting elements with an atomic number larger than 5 (carbon and heavier elements) and it is especially useful to retrieve information on spatial (2 dimensional) atom distribution. However, it may not be suitable for nanoparticles of complex composition, in complex matrices, and for large aggregates. Overall the method appears suitable, provided that sample preparation is standardised and reported in sufficient detail.

#### 3.1.3 **XPS** (X-ray photoelectron spectroscopy)

XPS is used to determine the elemental composition of nanomaterials' surfaces (see the surface chemistry section). Based on information from XPS analysis, it appears that XPS could also be a suitable method to characterise the chemical composition of uncoated materials such as metal oxides and CNTs, provided that the surface layer (0–10 nm) is representative for the core material; XPS was not evaluated in detail as a method for determining ‘chemical composition’ (OECD, 2016a).

#### 3.1.4 Methods not used in the Testing Programme

Many additional methods are available for compositional (elemental) characterisation of chemicals including nanomaterials, and a number of the commonly used methods are mentioned here. For substances with known crystalline structures, X-ray diffraction (XRD) is often a reliable method for identifying the main phases present (and by implication their chemical composition), although it is not so useful for quantifying stoichiometric variations or the presence of minor phases or trace elements. It is therefore best used in combination with more direct methods. For substances with one corresponding molecular formula the chemical composition may be confirmed by methods such as X-ray diffraction (XRD) and UV-VIS (Okitsu, 2013). Varieties of time-of-flight mass spectrometry (TOF-MS) (Tiede et al., 2008) can be used to analyse the composition of nanomaterials and provide information on the size of analysed particles when coupled with separation methods, e.g. Field Flow Fractionation (FFF). Aerosol time-of-flight mass spectrometry (ATOF-MS) (TSI, 2004) has been used to analyse the chemical composition of nanoparticles and simultaneously determine their size.

Some of the methods used to characterise organic substances in general can be also applied to NMs. Two non-destructive qualitative methods, Fourier transformed infrared spectroscopy (FTIR) and Raman spectroscopy (Carey, 2006), can be used to obtain information about organic and inorganic structures; in some cases they can be quantitative. Especially Raman spectroscopy, but also FTIR, are commonly used to characterise carbon based NMs, for example CNT (Branca et al., 2004) and graphene (Dresselshaus et al., 2010). FTIR has also been applied for characterisation of nanoparticles of metals (e.g. Au), metal oxides (e.g. ZnO) and quantum dots (CdS) (Dablemont et al., 2008) functionalised with organic compounds. Raman spectroscopy can additionally provide information on phase changes (both amorphous and crystalline) or lattice stress in NMs (Challa and Kumar, 2012).

Organic materials and the presence of organic molecules in NM are much less investigated than inorganic nanomaterials. For organic nanomaterials a technique that is often used is elemental analysis based on combustion (CHN(S) combustion analysis) that can successfully be employed for analysis of certain materials with high carbon content. However materials with high thermal stability, e.g. fullerenes and CNTs, could pose difficulties in the combustion process.

### 3.2 Surface chemistry (surface composition)

Surface chemistry here means the chemical nature of the surface, i.e. its composition in terms of elements and functional groups. A specific surface chemistry may be obtained through one or more treatment steps under well-defined conditions, without knowing exactly the resulting surface composition. The chemical composition of the particle surface is regulatory relevant information for nanomaterials (ECHA, 2017b, US-EPA, 2017, SCCS, 2012). Nanomaterials and nanostructured materials have a much larger specific surface area (SSA) than bulk materials. Therefore any interaction between the surface and its surroundings would be more relevant for nanomaterials than for bulk materials, including interactions arising from a specific chemical composition of the NM surface. The surface can be coated, e.g. to prevent dissolution, and/or functionalised. The dossiers’ “Surface chemistry” parts focus on the chemical (elemental) composition of the particle surfaces, rather than on analysis of functional groups. Some of the techniques used for the analysis of the composition of the entire particles like e.g. UV-VIS or FTIR can also be applied to characterise the surface chemical functionality.

In the Testing Programme the following methods were applied, see also Table 1:

#### 3.2.1 **XPS** (X-ray photoelectron spectroscopy)

XPS was applied to SWCNT, MWCNT, CeO_2_, ZnO and SiO_2_ and seems to be the only method used widely in the Testing Programme for this endpoint. XPS is commonly used to determine the elemental composition of nanomaterials' surfaces (Grant and Briggs, 2003), measuring the composition of a 0–10 nm surface layer, and XPS could be a suitable method to characterise ‘surface chemistry’ (OECD, 2016a) for materials that are stable under ultrahigh vacuum as required by XPS.

#### 3.2.2 **EDX (**or **EDS)** (energy dispersive X-ray), **SEM-EDX** (scanning electron microscopy - energy dispersive X-ray)

EDX was applied to nanoclays. SEM-EDX in combination with ICP-OES was applied to TiO_2_. See also the EDX section under Chemical composition.

#### 3.2.3 **ToF-SIMS** (time-of-flight secondary ion mass spectrometry)

Secondary ion mass spectroscopy (SIMS) is a technique applied in analysing the composition of solid surfaces and thin films, and is considered a very sensitive qualitative technique (in the range of ppm to ppb). In (static) SIMS the elemental, isotopic or molecular composition of the surface can be analysed to a depth of 1–2 nm. ToF-SIMS was applied to CeO_2_ and to ZnO. With this technique it is difficult to distinguish between properties of the particle core and of the surface, and furthermore the sample mounting (on a carbon tape) limited the analysis of C and Si in the sample. By principle ToF-SIMS is not quantitative, and it is rather useful to identify traces, but not to quantify them nor to compare samples.

#### 3.2.4 **RBA** (Rose Bengal adsorption method)

TiO_2_ was tested with this approach, by which the hydrophilicity of the surface can be determined from the Rose Bengal partitioning method (Müller, 1997) and binding constant can be calculated (Scatchard, 1949).

#### 3.2.5 Methods not used in the Testing Programme

The surface composition of nanomaterials can be also analysed by Auger electron spectroscopy (AES) (Briggs and Seah, 1983, Grant and Briggs, 2003) that can provide information on the elemental composition of a sample in the 2–20 nm depth range. It requires ultra-high vacuum, which is a major limitation and restricts its applicability to materials that are stable under these conditions, e.g. metals, metal oxides and CNTs (Ohdaira et al., 2002).

Electron energy loss spectroscopy (EELS) can also provide valuable information on the NM surface chemistry (Egerton, 2011). Similarly, X-ray fluorescence spectroscopy (XRF) can be applied for chemical composition analysis and can identify a wide range of elements from sodium to uranium with detection limits (LOD) in the ppm range; the LOD for XRF is however orders of magnitude higher than e.g. that of ICP-MS. Samples do not need any particular treatment before analysis, except removal of surface contamination. The analysis can be performed under vacuum conditions to allow detection of light elements. The resolution of the method depends on how X-ray fluorescence, is induced, e.g. irradiation of the sample by X-rays (usual) or electrons. A modification of this technique, Total Reflection X-ray fluorescence (TRXF) spectroscopy, is currently widely used in the electronics industry for quality control and an ISO technical specification is available (ISO, 2015b). Compared to XRF the TRXF method can reach detection limits in the low ppb levels or even below 1 ppb for some elements.

## 4 Physical characterisation

### 4.1 Particle size and particle size distribution

The particle size and particle size distribution are highly relevant in a regulatory context as their value define whether a given material is regarded as a nanomaterial in a regulatory context or not (e.g. NICNAS, 2009, Health Canada, 2011, EC, 2011, US-EPA, 2015, ISO, 2015a, US-EPA, 2017, EC, 2009, EC, 2012). Measuring particle size and size distribution would be the first step for physico-chemical characterisation of nanomaterials.

There is one OECD TG addressing these parameters, TG 110 “Particle Size Distribution/Fibre Length and Diameter Distributions” (OECD, 1981b), but it is not applicable to nanomaterials (OECD, 2009), and was thus not used in the Testing Programme. In the EU TG 110 is complemented by a Guidance Document (EC, 2002), but it is not designed to specifically address nanomaterials. The WPMN has recently (2016) initiated an activity to adapt TG 110 to make it applicable to nanomaterials.

It is well established that there is no single technique that can be applied to characterise the size of all nanomaterials (e.g. Linsinger et al., 2012). Consequently several techniques, based on different physical phenomena, are needed to measure the size range from nanometre-to micrometre-sized particles, and utilising a combination of methods for the determination of size and size distributions is often advantageous (e. g. based on particle imaging, sedimentation phenomena or light scattering) (NanoDefine, 2016, Gilliland et al., 2014) In the EU regulatory framework the use of more than one method (one being electron microscopy based imaging) for determination of size parameters for nanomaterials safety assessment has been recommended (SCCS, 2012, EFSA, 2011). For particulate nanomaterials the application of different methods and the interpretation of results are relatively straightforward, noting that the particle shape plays a significant role in the interpretation of results. Fibrous nanomaterials pose particular challenges as they have a high aspect ratio and may be entangled, and for example application of techniques that measure hydrodynamic size would not lead to a useful size distribution. Each method used to determine particle size and size distribution has its particular advantages and limitations, and multiple techniques should be used whenever possible in order to develop a complete understanding of the material (Linsinger et al., 2012).

Methods to determine the particle size distribution in a sample can be grouped according to whether particles are measured individually or as an ensemble (Stintz et al., 2010). They can be combined with fractionation techniques, where an external force/process is applied to separate the particles according to size. Subsequently an individual or ensemble detection technique is applied to each size fraction to determine the particle amount, e.g. the number or weight of the particles, to obtain a size distribution.

Ensemble techniques measure many particles in the sample at the same time and the size distribution is extracted from the combined signal of the measured particles. In general, ensemble techniques give statistically relevant results as they refer to many particles, but they may have insufficient resolution and size-dependent sensitivity. The combination of fractionation techniques and ensemble methods can overcome problems of resolution while retaining statistical relevance.

In counting techniques the size of individual particles is measured and counts of similar-size particles are placed into “size bins” to construct a size distribution. The counting methods overcome the problems of resolution, but require time and resources to obtain statistically relevant data.

The different techniques for size determination require that the nanoparticles are in a certain state so that they are accessible to the measurement. Particulate nanomaterials, including fibrous nanomaterials, are available in two main states: as dry powders or in suspension. Usually the sample requires some preparation and the choice of characterisation techniques is in part determined by this. To obtain reliable particle size distribution data, some techniques require for example that the particles of the sample, which should be representative of the material investigated, are well dispersed in a medium. Furthermore, as nanoparticles in general are prone to aggregate and/or agglomerate, the selected method must reliably cover a size range (far) beyond 100 nm to assure that the performed size-distribution analysis is representative for the material. The particle shape is an additional factor relevant for the size determination. The physical principle underlying the measurement technique often produces only an equivalent spherical particle diameter. However, most nanoparticles are not perfectly spherical and may have (very) irregular shapes. It is therefore necessary to state which actual measurand was used to determine the size, how the measurement results were evaluated and which quantity is reported. The output reported from the measurement techniques often corresponds to a characteristic quantity (e.g. Feret (min, mean or max) diameter or the maximum inscribed circle) or an equivalent size, e.g. equivalent spherical diameter. Thus for size measurement, it is very important to associate the result with the applied measurement techniques, provide the standard operating procedures (SOPs) for sample preparation and measurement, and describe precisely what is reported.

For monodisperse nanomaterials it is relatively straightforward to obtain and measure a representative sample, whereas polydisperse materials require measuring of a larger number of particles to determine the true particle size distribution. In general, reproducibility of particle size distribution measurements can be improved by standardising e.g. sample preparation and measurement conditions, and computational analysis.

In the Testing Programme the following methods were applied, see also Table 1:

#### 4.1.1 **DLS** (dynamic light scattering)

DLS was applied to C_60_, SWCNT, MWCNT, Ag, Au, TiO_2_, CeO_2_, ZnO, SiO_2_ and dendrimers. It was the most commonly used method in the Testing Programme, and an ISO standard is available (ISO, 2017b). DLS is a non-destructive ensemble technique and often used to measure particle size distribution, but in mixtures and polydisperse samples it can greatly underestimate the quantity of smaller particles. It is reliably applicable only to stable particle suspensions of monomodal and relatively narrowly dispersed size distributions (Calzolai et al., 2011). It is also fundamentally depending on the dispersibility and hence the state of dispersion of the sample. Therefore, besides the peak maximum value, also the polydispersity index should be given in the results. Data obtained by DLS are usually evaluated based on the assumption that the particles are spherical and the result is the equivalent hydrodynamic particle diameter. Therefore the shape plays a major role in the interpretation of the results and also other methodological issues were identified. DLS cannot distinguish between individual particles and aggregates/agglomerates (Calzolai et al., 2011), but it may still be useful. Other names for DLS are quasi-elastic light scattering (QELS) and photon correlation spectroscopy (PCS).

#### 4.1.2 **CLS** (centrifugal liquid sedimentation)

CLS was applied to CeO_2_, ZnO, SiO_2_ and nanoclays. It is a particle density-based ensemble technique that also depends on the dispersibility and hence the state of dispersion of the sample. CLS provides information on the Stokes diameter of the particles, as the data is usually evaluated assuming that the particles are spherical. Therefore the shape plays a major role in the interpretation of the results. CLS cannot distinguish between individual particles and aggregates/agglomerates. Several ISO CLS standards exist (ISO, 2001, ISO, 2007a, ISO, 2004) which could be applicable also to nanomaterials.

#### 4.1.3 **EM** (electron microscopy) including **TEM** (transmission EM) and **SEM** (scanning EM)

TEM was applied to C_60_, MWCNT, Ag, Au, TiO_2_, ZnO, TiO_2_ and SiO_2_; High Resolution (HR) TEM was applied to TiO_2_, and SEM was applied to SWCNT, MWCNT, CeO_2_ and ZnO.

EM is considered as the most useful and appropriate technique for direct investigation of NMs despite some disadvantages of traditional EM methods, which operate in high-vacuum thus being applicable only to solid samples. In addition to particle size, the particle size distribution may be obtained by EM techniques by measuring the size of many particles and counting them. Depending on the polydispersity of the material, a high number of particles need to be sized to ensure statistical reliability, and thus EM may not work well at environmentally relevant concentrations. Polydisperse materials also need a longer beam time. Furthermore, EM is not applicable to vacuum or electron beam sensitive materials (e.g. organic or biological etc.). Recently, specialised procedures and equipment have been developed which can, to a certain extent, overcome some of the limitations of working under vacuum conditions, but are not routinely used nor universally applicable. A main disadvantage of EM is that it provides 2D projections of 3D particles, i.e. it does not provide information on the size of all three dimensions of a particle. This is problematic especially for the size analysis of platelets, which often may be classified as nanomaterials only due to their small thickness.

Both SEM and TEM require samples of particles that are uniformly deposited with minimal particle overlap. TEM was found to be suitable for most nanoparticles, provided that they are not affected by the vacuum or electron beam used and are not agglomerated on the grid. TEM was found to give very good size and shape description of the materials including evaluation of agglomeration and aggregation; and a new semi-automatic method for size distribution analysis was proposed (de Temmerman et al., 2012, de Temmerman et al., 2014). SEM has similar restrictions as TEM but it is less accurate for small nanoparticles. For SEM the sample preparation is easier than for TEM. A limitation of SEM is that it provides information on conductive materials only, which can potentially be overcome by e.g. sputtering a thin layer of conductive material, such as silver or gold, on the sample. However, this may lead to changes of the material and the added layer increases the size of particles. Another way to resolve the conductivity issue for SEM is to operate under low voltage/current conditions, though this leads to a significant reduction of image resolution.

#### 4.1.4 Laser diffraction

Laser diffraction is a light scattering method that was applied to MWCNTs and the method was not considered particularly useful for small nanomaterials, but more suitable for larger materials, i.e. primarily above 50 nm (OECD, 2016a). Suitability depends on the relative refractive index and instrument employed.

#### 4.1.5 **UV-vis** (ultra-violet – visible analysis)

UV-vis was applied to Au for measurement of agglomeration/aggregation and to Ag for size measurements. However, peak position in UV-Vis spectroscopy may indicate particles size for plasmonic materials (only Ag, Au) but this is relevant only if the particles are monodispersed and perfectly spherical.

#### 4.1.6 **DOSY-NMR** (diffusion ordered spectroscopy nuclear magnetic resonance)

DOSY-NMR seeks to separate the NMR signals of different species according to their diffusion coefficient. In general, the NMR method is relevant for chemical and structural analysis of organic NMs (analysing magnetic nuclei such as ^1^H, ^13^C, ^19^F and ^31^P) and can detect the presence of monomers and contaminants in organic polymeric nanoparticles. In addition, in some cases it can also provide information on the surface functionalization and on the kinetics of functional group release (Wilson et al., 2015). It allows an accurate identification of the position of individual atoms in a given chemical structure elucidating their chemical environment and the surrounding elements. Given the presence of suitable nuclei, i.e. nuclei with a non-zero nuclear magnetic moment, this technique can be used both with organic and inorganic and hybrid NMs in solid state or in dispersion (Torres and Bottari, 2013, Roming et al., 2008). DOSY-NMR has high detection limits, necessitating high particle concentrations (in the range of g/L). Furthermore, the outcome is influenced by the composition of the particles and depends on field strengths and other factors. Although it is easily done for small dendrimers, it is much harder for larger materials, and also not suitable for a wide range of solid particle types (e.g. paramagnetic materials and those that would result in complex field modifications). Due to the specialised setup, this technique is more useful for research and development purposes than for routine industrial use. Its applicability is limited to some types of nanomaterials only (e.g. small dendrimers, which are organic materials).

#### 4.1.7 **SMPS** (scanning mobility particle sizer)

SMPS is a technique for measuring aerosols; it was applied to Ag, TiO_2_, CeO_2_, and ZnO; for Au it was used in exposure assessment and no information on size was given. SMPS provides information on the size of particles, agglomerates and aggregates. The model associated with SMPS is based on the assumption that the particles are spherical, and thus non-spherical particles or aggregates are expressed as their equivalent sphere (Wiedensohler et al., 2012). It may have fewer difficulties with agglomerated nanoparticles than most other methods.

#### 4.1.8 **AFM** (atomic force microscopy)

AFM was applied to Ag and SiO_2_. Atomic force microscopy (AFM) (Grobelny et al., 2011) is a microscopy technique that can be applied to size measurements, and as it gives 3D information on the particles also shape can be discerned; the accuracy of the 3rd dimension (thickness) is considerably better than the two lateral dimensions. It is one of the scanning probe microscopy (SPM) techniques (Samori, 2006).

#### 4.1.9 **(U)SAXS** ((ultra)small angle X-ray scattering)

(U)SAXS was applied to TiO_2_, SiO_2_ and Ag and is described in the section on specific surface area characterisation. (U)SAXS cannot be used as a stand-alone technique for size determination, but works well for confirming results from other techniques.

#### 4.1.10 Methods not used in the Testing Programme

Some other methods are listed below; as the method development is intense the list is not exhaustive.

A microscopy technique that can be applied to size (and shape) measurements is scanning probe microscopy (SPM) (Samori, 2006), which includes AFM (see above) and scanning tunnelling microscopy (STM) (Marchini et al., 2007) and variations thereof. STM images may give directly the 3D morphology of certain complex samples (e.g. CNT (Terrones et al., 2004)) and can resolve both their electronic density and atomic structure simultaneously under appropriate working conditions.

Multi-angle light scattering (MALS) is also a commonly used technique that measures particle size in liquid dispersions (Xu, 2000), providing information on the geometric diameter. MALS is a commonly used technique for characterisation of proteins and polymers and it can be applied to non-spherical nanoparticles; it is often used in combination with FFF (field flow fractionation).

WAXS (wide angle X-ray scattering) (Graewert and Svergun, 2013) is another technique that employs light scattering and can be used for size distribution analysis.

Fractionation techniques have been combined with detection techniques to determine the size distribution of particles (NanoDefine, 2016). FFF techniques are very powerful chromatographic methods and can, in principle, separate particles from 1 nm to several micrometres, regardless of their chemical composition. FFF separation techniques provide a means to analyse the particle size distribution in very complex mixtures, and the separated fractions of the injected dispersion can be collected for further physico-chemical analysis (Dubascoux et al., 2010). Another widely used chromatographic technique is capillary hydrodynamic fractionation (CHDF). It operates similarly to FFF methods; however the principle of particle separation is based on differences in transport rates in a capillary, related to their location in the eluent. CHDF can separate particles in the range 15–1000 nm and it can be coupled with different detectors (Philippe et al., 2014).

Single particle (sp) ICP-MS can provide information on size and size distribution of nanoparticles. In general sp-ICP-MS can be run on modified conventional ICP-MS instruments, which are however limited in terms of time resolution and detection efficiency; sample preparation is simple (often only dilution) and the measurement time per sample relatively short (1 min) which allows high throughput analysis. Furthermore, it is specific for a given chemical composition and provides number-based size distribution directly, however currently it is useful only for a quite limited number of particle types (Linsinger et al., 2014, ISO, 2017a).

### 4.2 Specific surface area

The (volume) specific surface area [(V)SSA] is relevant in a regulatory context, since if requested in specific legislation the parameter can be used to decide whether a given material is a nanomaterial (EC, 2011). Specific Surface Area is relevant information for nanomaterial registration in the USA (US-EPA, 2017), and it is recommended to be reported for nanomaterials used in EU e.g. in cosmetic products (SCCS, 2012), food and feed (EFSA, 2011) or biocidal products (ECHA, 2014). As nanomaterials can have a much larger (V)SSA than corresponding bulk materials, any interaction of the surface with its surroundings would be stronger for nanomaterials.

The OECD is currently considering whether a new Test Guideline or Guidance on how to measure surface area (BET etc.) could/should be developed.

In the Testing Programme the following methods were applied, see also Table 1:

#### 4.2.1 **BET** (surface area calculated from gas adsorption, based on the Brunauer-Emmett-Teller method)

BET (Brunauer et al., 1938) was applied to C_60_, SWCNT, MWCNT, Ag, Au, TiO_2_, CeO_2_, ZnO, SiO_2_ and nanoclays to determine the specific surface area. BET is suitable for nanomaterials that do not absorb the gas used. An important limitation is that it is applicable only to dry solid samples (it is thus not applicable to dendrimers), while toxicological testing often involves the use of liquid suspensions. For microporous solid nanomaterials specific adaptations of BET are needed, and the technique is not optimal. ISO has published a standard on the determination of the specific surface area of solids by using BET (ISO, 2010).

#### 4.2.2 **SAXS** (small angle X-ray scattering)

SAXS was applied to TiO_2_ and SiO_2_. It is not recommended as a primary method to determine specific surface area. However, it can be used to measure the specific surface area by relating the tail-end constant of the small–angle curve to the integral scattering of the material. SAXS is suitable for investigating biological samples and complex matrix materials, and is commonly used on biological material, for example cells and tissues, proteins and protein folding, colloids, micelles, bacteria and viruses, giving important information for subsequent toxicity testing of nanomaterials (Saw, 2005).

A new technique for determining volume specific surface area, Transmission Electron Tomography, based on TEM, was developed and investigated for SiO_2_ in the Testing Programme (van Doren et al., 2011). ISO has published a standard on SAXS (ISO, 2015c).

### 4.3 Porosity

Porosity is relevant in a regulatory context as the parameter is related to the inner surface of a material. For porosity, several pore sizes are distinguished: macropores, mesopores and micropores, and the methods to measure the different types differ. OECD concluded (OECD, 2014a) that current methods to measure porosity are not nanospecific but may be applicable or adaptable. Standardised measurement methods have been proposed by ISO (e.g. ISO, 2006, ISO, 2007c, ISO, 2016a).

In the Testing Programme the following methods were applied, see also Table 1:

#### 4.3.1 **Gas adsorption method**, modelled by **BET** (Brunauer-Emmett-Teller theory) or **BJH** (Barrett-Joyner-Halenda theory)

For measuring porosity, BET was applied to TiO_2_, CeO_2_, ZnO and SiO_2_ and BJH (Barrett et al., 1951) was applied to ZnO and CeO_2_. The use of BET and BJH for determining porosity is standardised by (ISO, 2006) for non-microporous materials. For microporous materials, i.e. materials with pores of a diameters less than 2 nm, microporosity should be taken into account using e.g. a different ISO standard (ISO, 2007c).

#### 4.3.2 Mercury Intrusion Porosimetry

Mercury Intrusion Porosimetry (Powers et al., 2006) was applied to C_60_, SWCNT, and MWCNT. The high pressure needed for measurement may deform the material tested, and when applied to metals, amalgams may form and thus the method is not suitable for metal-containing nanomaterials. For small pores it has similar limitations as BET/BJH.

The interpretation of results for BET, BJH and Mercury Intrusion Porosimetry strongly depends on the pressure and temperature during the test, and on the model used to deduce porosity from the adsorption data.

#### 4.3.3 Methods not used in the Testing Programme

Though not applied to measure porosity in the Testing Programme, also **SAXS** can provide information on porosity and pore size distribution as well as on size, shape, inter-particle correlations and density fluctuations of nanostructures (Saw, 2005).

### 4.4 Crystallite size and crystalline phase

Crystalline phase, and for crystalline materials also crystallite size, are a regulatory relevant endpoints (e.g. SCCS, 2012, ECHA, 2017a, EFSA, 2011) that further characterise the material. It is well known that materials with the same composition but different crystalline structure may exhibit quite different toxicological or reactivity profiles (e.g. SiO_2_, TiO_2_).

In the Testing Programme the following methods were applied, see also Table 1:

#### 4.4.1 **XRD** (X-Ray diffraction)

XRD, which was applied to Ag, Au, TiO_2_, CeO_2_, ZnO SiO_2_ and dendrimers, is a generally accepted characterisation method. It was evaluated to be a very suitable method to determine crystallinity of nanomaterials. Average crystallite sizes (not necessarily the particle sizes) can be calculated from XRD measurements via the Scherrer equation for sizes below 100–200 nm. XRD of SiO_2_ was used to confirm that the SiO_2_ tested was amorphous; no size determination is possible for amorphous materials.

#### 4.4.2 Raman spectroscopy

Raman spectroscopy is commonly used in chemistry to provide a fingerprint by which molecules can be identified. It was applied to SWCNT and MWCNT for which the method can provide information about bonding structure and amount of non-amorphous material.

#### 4.4.3 Electron microscopy

TEM was applied to C_60_, MWCNT, Ag and Au. Although crystalline structures can be seen at very high magnifications (atomic resolution) in electron microscopy, according to the evaluating experts determination of crystallite size and crystalline phase by EM is considered as ‘pushing the method’, and would require significant beam time to measure a (statistically sufficient) number of particles (OECD, 2016a).

#### 4.4.4 SAXS

SAXS was applied to TiO_2_ and SiO_2._ SAXS can provide information about the shape and size of nanoparticles, characteristic distances of partially ordered materials, pore sizes, and many other data. SAXS can deliver structural information of nanoparticles and is commonly used to confirm that materials are amorphous or non-homogeneous.

#### 4.4.5 Differential scanning calorimetry (DSC)

DSC was applied to dendrimers. It is a general method for investigating phase transitions and other temperature dependent phenomena, and can also be applied to nanomaterials. It does not give information on the type of crystallinity or size of crystals. The possibility to use DSC to elucidate phase transitions is included in OECD TG 103 (Boiling point) and TG 102 (melting point/melting range).

#### 4.4.6 Methods not used in the Testing Programme

For crystallinity characterisation also electron and neutron scattering techniques can be employed; but they are less commonly used due to their higher cost.

In addition, in destructive methods based on heating the material such as Differential Thermal Analysis (DTA) the studied material and an inert reference undergo identical thermal cycles, while recording any temperature difference between sample and reference. The differential temperature is then plotted against time or temperature. Changes in the sample, either exothermic or endothermic, can be detected relative to the inert reference. A DTA curve thus provides data on the transformations that have occurred, e.g. glass transitions, crystallization, melting and sublimation.

Furthermore, WAXS, a variant of X-ray scattering, can resolve even smaller dimensions and is used for analysing highly ordered or crystalline structures.

### 4.5 Shape

Though not listed as a parameter in the set of additional endpoints proposed for nanomaterials, shape is an important particle descriptor. Within the EU, new guidance defining “nanoform” for REACH (EC, 2006) registration was published by ECHA and shape is one of the key parameters for “nanoform” definition (ECHA, 2017b). US EPA also considers shape and morphology of the particles relevant information for registering and distinguishing discrete nanoscale forms of specific chemical substances (US-EPA, 2017). Shape determination is also required for the safety assessment of nanomaterials in the EU for the use in e.g. cosmetics (SCCS, 2012) or food and feed products (EFSA, 2011). Furthermore, some plasmonic materials have optical properties which are related to shape but these are a limited number of special examples.

Many particulate materials do not have well-defined, uniform shapes such as spheres or rods, and the particle shape within the material varies significantly. Thus, for many materials the concept of shape is not easily quantifiable which makes the assessment of shape a challenging issue. Furthermore, a number of methods for e.g. particle size determination give results based on the assumption that the particles are spherical, and thus it is important to be aware of the real particle shape(s) in order to correctly interpret measurements. Many shape descriptors have been devised, attempting to quantify shape (e.g. de Temmerman et al., 2012), including sphericity, convexity, aspect ratio and fractal dimension (Klobes et al., 2006). An ISO standard on image analysis of shape exists which includes standardised shape factors (ISO, 2008). This, and other (national) standards, should be considered when defining the particle shape (ISO, 2013a).

For nanomaterials, EM is one of the few techniques that have a sufficient resolution to provide reliable images and thereby information on the shape of the nanoparticles. As already mentioned, EM provides a 2D projection of 3D particles and care should be taken to avoid bias due to preferential orientation effects. It is especially important when analysing platelet-like particles as their smallest dimension is often not well accessible for EM and hence can remain undetected. Although there are approaches to standardise reporting of shape data (ISO, 2008), a number of different protocols, i.e. ways of expressing the relevant dimension, are still used. AFM and STM provide 2.5D-images of particles, however, shape analysis using these techniques is prone to errors due to tip effects, usually time consuming and thus expensive. They seem to be difficult to adapt to routine application.

The OECD recognises the shape/aspect ratio as an important parameter for physical identification of nanomaterials (OECD, 2016b), as well as an influencing factor in determining the possible adverse effects of nanomaterials. It has recommended the use of ISO definitions for shape descriptors (e.g. ISO, 2008). OECD has also suggested developing guidance on when to apply which shape descriptor.

## 5 Fate characterisation

### 5.1 Octanol-water partitioning coefficient

The octanol-water partitioning coefficient was originally listed by the WPMN for its Testing Programme, but later it was concluded that the parameter is meaningless for particulate, insoluble nanomaterials. Whereas it would be relevant to be able to measure relative hydrophobicity/hydrophilicity, no generally accepted methods were identified for nanomaterials. For particles that quickly dissolve into ions or individual molecules usually the same approach applies as for conventional chemicals. The octanol-water partitioning coefficient will not be further described or discussed here.

### 5.2 Water solubility/dispersibility

Water solubility is often considered as one of the factors determining the distribution of nanomaterials, e.g. environmental fate. As a consequence it is also considered as relevant for exposure and hazard patterns. Therefore also for nanomaterials, the endpoint is considered to be relevant and required in regulatory contexts (e.g. REACH, Annex VII, 7.7 (EC, 2006)). Moreover in EU for certain product specific regulations water solubility is indicated as a key parameter for the material classification as nanomaterial or not (EC, 2009) and in principle it is commonly required in the safety assessment of nanomaterials used in consumer products (e.g. SCCS, 2012, EFSA, 2011, EC, 2012). For particulate materials, however, dissolution and dissolution rate are more relevant parameters. Fully dissolved materials, which are present as ions or individual molecules, are addressed by the approach applied to conventional chemicals. As for chemicals in general, the dissolution of nanomaterials depends also on the solvent and has to be determined for each solvent-solute combination, and furthermore, dissolution may be affected by many other factors such as temperature, impurities concentration or forces between particles. For (environmental) fate and toxicity studies it could be important to have information on the kinetics of dissolution or dispersion of the nanomaterial as it may change with time and therefore it may modify the toxicity profile of a nanomaterial over time. For nanomaterials, it is furthermore important to distinguish between dissolution, which occurs at molecular or atomic/ionic levels, and dispersibility, which occurs at particle levels. In addition, there is a fundamental difference between (water) solubility where individual molecules or ions are released into the surrounding fluid, and dissolution (in water) where chemical reaction(s) between the (nano)material and water cause (newly formed) molecules or ions to be released into the surrounding fluid.

OECD TG 105, which addresses water solubility, was evaluated to not be applicable for nanomaterials (OECD, 2009, OECD, 2014b) and was proposed as a candidate for updating (OECD, 2016b). Especially for nanomaterials that disperse into small primary nanoparticles, the elution method needs to be adapted with appropriate particle detection methods.

Many of the techniques which are used to determine size and size distribution (e.g. DLS) are also suitable for measuring changes in the state of dispersion of a system, which implies quantification of both primary particle size distribution (un-agglomerated) and the agglomerate/aggregate size distribution of the system.

In the Testing Programme the following methods were applied, see also Table 2:

**Table 2:** Physico-chemical parameters for fate characterisation of nanomaterials and suitability of associated analytical methods.

Methods a	Available standards	Tested in OECD WPMN b	Suitable for testing nanomaterials?	Remarks
**Water solubility and Dispersibility** Water solubility is a key factor in (environmental) fate and distribution. For nanomaterials, it is important to distinguish between solubility and dispersibility.				
Flask method	OECD TG 105	Au, CeO_2,_ Dendrimers	No	The method needs adaptation to nanomaterials, and it does not distinguish between solubility and degradation.
Turbidity	ISO 7027: 2016	CeO_2_, ZnO, Nanoclay	Yes, with restrictions	May be used as a first screening method. The applicability of the method to nanomaterials in general is unknown.
Spectrophotometry (Motomizu method)	–	SiO_2_	Not evaluated	
Equilibrium dialysis	–	Ag	Not evaluated	
Other methods and environmentally relevant media	–	MWCNT, Ag, TiO_2,_ CeO_2_, ZnO, SiO_2,_ Nanoclay	Not evaluated	
**Aggregation/Agglomeration** Aggregation/Agglomeration is highly relevant for exposure, fate and hazard assessment. It is also relevant for dispersion stability, which influences test accuracy.				
DLS	ISO 22412:2017	Ag, Au, TiO_2,_ CeO_2_, ZnO, SiO_2,_ Dendrimers	Yes, with restrictions	Results are influenced by particle shape and dispersibility. Cannot distinguish between individual particles and aggregates/agglomerates. In mixtures and polydisperse samples DLS can greatly underestimate the quantity of smaller particles.
CLS	ISO 13318-1:2001; ISO 13318-3:2004 ISO 13318-2:2007	ZnO	Yes, with restrictions	Results are influenced by particle shape and dispersability.
SEM	CEN/TC 352 (under development)	SWCNT, MWCNT, CeO_2_, ZnO, Nanoclay	Yes, with restrictions	Applicable to materials that are stable in high-vacuum conditions. May not work well at environmentally relevant concentrations. Provides 2D-projections of 3D-particles and aggregates/agglomerates, thus less suitable for non-spherical particles (e.g. platelets). Risk of agglomeration on the grid. Less sensitive than TEM, but easier sample preparation.
TEM	CEN/TC 352 (under development); ISO/TC 229 (under development)	C_60_, SWCNT, MWCNT, Ag, Au, TiO_2,_ ZnO, SiO_2,_ Dendrimers	Yes, with restrictions	Applicable to materials that are stable in high-vacuum conditions. May not work well at environmentally relevant concentrations. Provides 2D-projections of 3D-particles, thus less suitable for non-spherical particles (e.g. platelets). Risk of agglomeration on the grid.
Laser diffraction	–	SiO_2,_	No	
UV-Vis	–	Au	No	
DOSY-NMR	–	Dendrimers	No	
AFM	–	SiO_2_	Yes, with restrictions	Gives 2.5D information.
**Zeta potential** Zeta potential highly influences dispersion stability, which is relevant for exposure and fate and test accuracy. Zeta potential not only depends on intrinsic particle properties but also on environmental conditions (such as pH).				
ELS	ISO 13099-2: 2012	C_60_, SWCNT, MWCNT, Ag, TiO_2,_ SiO_2,_ Dendrimers	Yes, with restrictions	Suitable for nanomaterials that can be dispersed in a liquid. Not suitable for hydrophobic nanomaterials in aqueous media, or for application in high conductivity media.
Electro-acoustic phenomena measurements	–	–	Not evaluated	
**Dustiness** Dustiness is relevant for exposure assessment, especially at the workplace. Dustiness not only depends on intrinsic particle properties but also on environmental conditions (such as temperature and humidity).				
Rotating drum	EN 15051	CeO_2_, ZnO	Yes, with restrictions	Provides results on health-relevant dustiness but does not distinguish between different particle sizes; mass-based; has limited suitability for nanomaterials.
Small rotating drum	–	TiO_2,_ SiO_2_	Yes	Downscaled method of the EN 15051 rotating drum (using much smaller quantities of material), not yet standardised.
Continuous drop method	EN 15051 DIN 55992-2	MWCNT, CeO_2_	Yes, with restrictions	Less suitable for powders that are sensitive to caking and for fluffy powders.
Vortex shaker	–	C_60_, SWCNT, MWCNT, TiO_2,_ SiO_2,_	No	Although the method is considered simple and compact, it does not provide health-relevant dustiness.

a For abbreviations and technical details of methods see Supplementary Information.

b Analytical methods have been evaluated for measurement of physico-chemical properties of specific nanomaterials within the OECD WPMN Testing Programme of Manufactured Nanomaterials as indicated.

#### 5.2.1 Flask method

The flask method, which is part of OECD TG 105, was applied to Au (treated to be hydrophilic and remain in the water phase) and CeO_2_, concluding that TG 105 needs to be adapted to have more relevant cut-off values when removing undissolved particles. It was also noted that TG 105 does not allow distinguishing between solubility and degradation (by chemical reaction) for normal chemicals, neither does it address issues relating to hydrophilicity, hydrophobicity or organic materials. The results of the method therefore must be interpreted taking all properties of the test material into account. OECD has also suggested that the measurand of interest (beginning with a pre-determined unit of particles in a standardised solution and temperature) is the mass proportion of nanomaterials held in solution, and whether this mass diminishes after a set period of time; or to determine the time required for mass to diminish by X% (OECD, 2009) Currently used methods for solubility assessment of conventional chemicals could in principle be used for nanomaterials; but protocols and guidelines specifically tailored to nanomaterials are needed, and are under development (Tantra, 2016, Hartmann et al., 2015).

#### 5.2.2 Turbidity

Turbidity was used to determine dispersion stability/dispersibility for ZnO, CeO_2_, and Nanoclay. The turbidity method was found to give only qualitative size information. It may be used as a first screening method to be followed by more quantitative particle sizing measurements. Furthermore, the information available in the dossier was insufficient to evaluate the applicability of this method for nanomaterials in general. The updated ISO 7027:2016) (ISO, 2016d) standardises the technique, and the detection by nephelometers can also cover particles in the nanoscale range.

### 5.3 Agglomeration/aggregation

Different regulatory needs were identified by the WPMN that make information on agglomeration/aggregation (and dispersion) relevant (e.g. SCCS, 2012, EFSA, 2011). In principle, the state of dispersion of particulate systems indicates the degree to which particles are agglomerated. Different forces may hold particles together in groups or clusters and the most fundamental ones are attractive van der Waals forces and Coulomb forces. The strength of the van der Waals forces is related to the atomic properties of the surface atoms, shape (geometry) and distance of these surfaces from each other. The state of agglomeration strongly depends on the experimental conditions, e.g. medium and pH. The state of dispersion influences the result(s) of any test that requires the sample to be in liquid dispersion, as the particles will agglomerate and aggregate in suspensions when repulsive forces (electrostatic, steric effects) are not strong enough to keep the particles sufficiently separated to prevent short range attractive forces (Van der Waals) becoming dominant. The particles may also be stabilised by surface charge or stabilisers present (purposely or not) in the media. The determination of dispersion stability, which is the ability of a dispersion to resist changes in properties over time, is fundamental prior to any toxicity testing, especially if the nanoparticles have to be dispersed in a medium before administration. Changes in dispersion stability affect physical properties that in turn can affect the environmental fate and hazard properties of chemical substances including nanomaterials. Information on dispersion stability is also relevant information for nanomaterial registration and identification of “discrete forms” in the USA (US-EPA, 2017).

The parameter influences other properties, for example the fate and exposure assessment is affected by the degree of agglomeration/aggregation, and mobility is affected by the dynamic state of agglomeration and by dispersion stability. The WPMN noted that methods are needed, but not yet available, that can be used for in situ measurements of the dispersion/agglomeration/aggregation state in actual test systems. Furthermore, the state of agglomeration/aggregation is also relevant for hazard assessment, as test accuracy is influenced by dispersion stability, which is related to agglomeration/aggregation.

Particle size affects the dispersion state in two ways: for larger particles (including agglomerates and aggregates) sedimentation may occur, and for smaller particles the attractive van der Waals forces per particle are relatively stronger than for larger ones. Thus with decreasing size re-dispersion of agglomerated particles becomes more difficult. Gentle stirring is insufficient to re-disperse a strongly aggregated material, which requires major (controlled) energy input, such as grinding or sonication. The high energy input may modify the properties of the material.

Currently there is no OECD TG for agglomeration/aggregation state characterisation. OECD (OECD, 2016b) has expressed its interest in assessing the need for a Test Guideline on aggregation, and it was suggested to first evaluate the usefulness of the ISO technical report “Guidelines for the characterisation of dispersion stability” (ISO, 2013b) for OECD purposes.

Three projects on agglomeration/aggregation and dissolution are on-going within the OECD Test Guidelines Programme (TGP): developing a) a Test Guideline on agglomeration behaviour of nanomaterials in different aquatic media, b) a Guidance Document (Decision-Tree) on agglomeration and dissolution behaviour of nanomaterials in aquatic media and c) a Guidance Document on dissolution rate of nanomaterials in the aquatic environment. In addition the possible development of either a new Test Guideline or a Guidance Document on determination of aggregation/agglomeration status of NMs is under consideration.

In the Testing Programme, the methods described above in the section on “particle size and particle size distribution” were applied also to determine the agglomeration/aggregation, and the reader is referred to the section relating to electron microscopy (TEM and SEM), AFM, DLS, CLS, Laser Diffraction, SMPS, UV-vis, (U)SAXS and Dosy-NMR.

In general, when results are to be used for fate and transport modelling, aerosol or liquid based methods, reflecting environmental conditions, are better suited for determining agglomeration/aggregation than microscopy methods.

#### 5.3.1 Turbidity

This method is described above in the section on Water solubility/Dispersibility.

### 5.4 Zeta potential

The zeta potential is basically the difference in potential between the dispersion medium and a stationary layer of fluid attached to the dispersed particles, and it is related to the electrical charge on the surface of nanoparticles dispersed in liquid media that depends on the particle itself and the surrounding medium. The Zeta potential is not directly measurable and is thus calculated using Henry's equation applied to electrophoresis measurements. It is one of the main factors controlling the stability of a dispersion and it may thus have influence e.g. on the fate and behaviour of a nanomaterial in the environmental or on whether a NM can be tested in an aqueous suspension or not. Even small changes in the environment, such as a slight pH change, ionic strength or concentration of ligands, can considerably influence the aggregation rate of dispersed particles, and therefore it is common practice to measure the zeta potential as a function of pH as well as to determine the isoelectric point, i.e. the pH value at which the zeta potential is equal to zero. The Zeta potential is thus regulatory relevant information (e.g. US-EPA, 2017, SCCS, 2012, EFSA, 2011).

OECD experts (OECD, 2016b) suggested that zeta potential should be always reported at the pH intended to be used for the given test system, the model used should be identified and the IEP (Iso-Electric Point) should always be reported. As there is no OECD TG for zeta potential measurement, OECD is considering the possibility to develop a new TG on determination of Zeta potential for NMs.

Some ISO standards for determining zeta potential are available (ISO, 2000, ISO, 2012c, ISO, 2012d). In the Testing Programme the following methods were applied:

#### 5.4.1 **ELS** (electrophoretic light scattering)/electrophoretic mobility method

For C_60_, SWCNT, MWCNT, Ag, TiO_2_, SiO_2_, and dendrimers the zeta potential was calculated based on measurement of electrophoretic mobility.

The method was found to be suitable for a wide range of nanomaterials, but it is not suitable for hydrophobic nanomaterials in aqueous media, or application in high conductivity media (OECD, 2016b). ELS is standardised under ISO (ISO, 2012b) and is suitable for all nanomaterials that can be dispersed in a liquid.

#### 5.4.2 Methods not used in the Testing Programme

Also electro-acoustic phenomena measurements can be used to determine the zeta potential of dispersed particles.

### 5.5 Dustiness

Dustiness is defined as “the propensity of a material to generate airborne dust during its handling” (ISO, 2012b). Dustiness is not an intrinsic property, as in addition to the physico-chemical properties of the particles (size, shape, surface area, composition, aggregation etc.) also external factors may influence the dustiness of a material, e.g. its moisture content, as well as the environment (temperature, pressure, humidity). The results obtained for dustiness depend on the test method used and are measured in arbitrary units, and do not provide information on the number of particles or their size distribution nor on the particle shape. Dustiness is especially important in working environments (e.g. to set Occupational Exposure Limits), and it is thus regulatory relevant information. In EU it should be reported when registering substance under REACH (ECHA, 2016, ECHA, 2017a), as well as for safety assessment in certain product specific legislations (e.g. SCCS, 2012, EFSA, 2011).

There is no OECD TG for measuring dustiness. A European standard, EN 15051, (British Standards, 2013a) is available and contains two methods, the rotating drum (British Standards, 2013b) and continuous drop methods (British Standards, 2013c). Both methods require a rather large amount of material, typically more than 500 g. EN 15051 provides results on health-relevant dustiness, using mass-based protocols that do not distinguish between different particle sizes and do not clearly indicate presence or absence of particles smaller than 100 nm. The description states that EN 15051 has a limited suitability for nanomaterials. The two methods are relevant to powders in general, and some more work is needed to adapt them to nanomaterials.

In the Testing Programme the following methods were applied, see also Table 2:

#### 5.5.1 Rotating drum

In the Testing Programme the rotating drum method from EN 15051 was applied to CeO_2_ and ZnO. The method itself however states that it has a limited applicability to nanomaterials, and has been downscaled to make it more nano-applicable (see ‘small rotating drum’ below).

#### 5.5.2 **SD** (small rotating drum)

In the Testing Programme dustiness was measured for TiO_2_ and SiO_2_ by a downscaled EN 15051 rotating drum (the small rotating drum (SD) requires less than 6 g of material) method (Nanogenotox, 2012).

#### 5.5.3 Continuous drop method

The continuous drop method was applied to MWCNT and CeO_2_. The continuous drop tester is less suitable for powders that are sensitive to caking and for fluffy powders.

In addition DIN 55992-2 (DIN, 1999) was applied to MWCNT.

#### 5.5.4 **VS** (vortex shaker method.)

The vortex shaker method was applied to C_60_, SWCNT, MWCNT, TiO_2_ and SiO_2_ and is considered easy, simple and compact, but the resulting dustiness index is not representative for health-relevant dustiness.

As expected, the results from the SD and VS methods applied to SiO_2_ differ significantly due to the differences in the activation energy in the simulated handling. Currently CEN/TC 352 has launched activities to standardise and harmonise both methods for nanomaterials.

## 6 Characterisation of inherent reactivity

### 6.1 Redox potential

Redox potential, also called reduction potential or oxidation/reduction potential, is a measure of the tendency of a substance to acquire or release electrons and thereby respectively be reduced or oxidised. As the redox potential reflect reactivity of a substance it is relevant for regulatory safety assessment. As such it is a required endpoint in the registration of chemical substance in the EU (REACH, Annex VII, 7.13 (EC, 2006)) as well as in safety assessment of ingredients in cosmetics products (SCCS, 2012) and food and feed products (EFSA, 2011). There is no OECD TG for measuring redox potential.

In the Testing Programme the following methods were applied, see also Table 3:

**Table 3:** Physico-chemical parameters for characterisation of nanomaterial inherent reactivity and suitability of associated analytical methods.

Methods a	Available standards	Tested in OECD WPMN b	Suitable for testing nanomaterials?	Remarks
**Redox potential** As the redox potential reflects reactivity of a substance, it is relevant for regulatory safety assessment.				
SDR (Oxo-Dish method)	–	TiO_2,_ SiO_2_	No	The test endpoint is oxygen level and not redox potential; although dissolved oxygen may correlate with redox potential, this is not always the case.
Potentiometric method	–	ZnO, CeO_2_	No	Method is more sensitive to the ions in the test medium than to the added nanomaterials.
**Radical formation potential** The formation of reactive oxygen species (ROS) induced by free radicals is a relevant factor in the toxicity of nanomaterials.				
EPR/ESR	ISO/TS 18827	TiO_2_	Yes	Suitable for a wide range of nanomaterials, provided that they have unpaired electrons.
Potassium Iodide test (KI)	–	ZnO, CeO_2_	Yes, with restrictions	Suitable for nanomaterials that generate hydroxyl radicals.
Benzoic acid formation in phosphate buffer solution	–	SiO_2_	No	
**Photocatalytic activity** Photocatalytic materials generate ROS under irradiation of light.				
Rhodamine-B dye degradation	–	ZnO, CeO_2_	Yes, with restrictions	Not suitable for nanomaterials that form coloured suspensions.
DPPH degradation	–	ZnO, CeO_2_	Yes, with restrictions	Not suitable for nanomaterials that form coloured suspensions.
EPR	–	TiO_2_	Yes, with restrictions	There was insufficient information in the dossier to evaluate the applicability of this method to other nanomaterials.
Orange-II degradation	–	TiO_2_	Yes, with restrictions	There was insufficient information in the dossier to evaluate the applicability of this method to other nanomaterials.
Formaldehyde degradation	–	TiO_2_	Yes, with restrictions	There was insufficient information in the dossier to evaluate the applicability of this method to other nanomaterials.
Acetaldehyde degradation	ISO 22197:2	C_60_, SWCNT, MWCNT	Yes, with restrictions	Suitable for carbon nanomaterials and (metal)oxide forms of nanomaterials. Insufficient for quantitative measurements (may be improved by optimisation of the sample preparation).
Monitoring of photo-induced NADH	ISO (under development)	–	Not evaluated	

a For abbreviations and technical details of methods see Supplementary Information.

b Analytical methods have been evaluated for measurement of physico-chemical properties of specific nanomaterials within the OECD WPMN Testing Programme of Manufactured Nanomaterials as indicated.

#### 6.1.1 Potentiometric method

The potentiometric method measures the redox potential as the difference in potential across two electrodes (a Pt electrode against a double junction Ag/AgCl reference electrode), and was applied to suspensions of CeO_2_ and ZnO.

#### 6.1.2 Oxo-dish method

The Oxo-Dish method (sensor disk reader, SDR) monitors oxygen levels inside a 24-well plate during 24-h incubation using a fluorescence method. SDR was applied to SiO_2_ and TiO_2_.

An evaluation of the methods (OECD, 2016a) concluded that neither method is adequate to measure the redox potential for nanomaterials. The potentiometric method is more sensitive to ions in the test medium than to the added nanomaterials. The Oxo-Dish method was found unsuitable for measuring redox potential in nanomaterials, because the test endpoint is oxygen level and not redox potential. Although dissolved oxygen may correlate with redox potential, this is not always the case.

### 6.2 Radical formation potential

Radical formation potential is important because many studies indicate that the formation of reactive oxygen species (ROS) induced by free radicals seems to be a key event triggering some NM adverse effects, and it is thus highly relevant information to assess nanomaterials, also in a regulatory context. There is no OECD TG for measuring radical formation potential.

Within the Testing Programme the following methods were applied:

#### 6.2.1 Electron paramagnetic resonance

Electron paramagnetic resonance (EPR), also known as electron spin resonance (ESR), was applied to TiO_2_. ESR is regarded as a suitable method for a broad range of nanomaterials, provided that they have unpaired electrons as paramagnetic properties are due to the presence of these. ISO has standardised the EPR method for measuring reactive oxygen species (ROS) generated by metal oxide nanomaterials (ISO, 2016b).

#### 6.2.2 Potassium iodide test (KI)

The KI test is a method for assessing the general oxidative activity of nanomaterials under irradiation. KI was applied to ZnO and CeO_2_. It was found suitable for nanomaterials that generate hydroxyl radicals.

#### 6.2.3 Benzoic acid phosphate buffer solution (PBS)

PBS was applied to SiO_2_. Using benzoic acid in a phosphate buffered hydrous solution (PBS) was found unsuitable for the evaluated nanomaterial, synthetic amorphous SiO_2_, as the results reported in the dossier may have been an artefact of the sample preparation and analysis conditions.

A general question for future consideration is which specific radicals should be measured to identify the radical formation potential of a nanomaterial.

### 6.3 Photocatalytic activity

The photocatalytic activity of materials refers to their ability to create electron-hole pairs under irradiation of light, which then generate reactive oxygen species (ROS), for example free radicals (e.g. in aqueous solution or in presence of O and H sources, superoxide radicals, hydroxyl radicals, etc.), hydrogen peroxide, and singlet oxygen (see also the previous section on Radical formation potential). The lifetime of ROS is generally below a millisecond, which makes ROS detection challenging. Most materials are not photocatalysts, and in a regulatory context the information is relevant on a case-by-case basis. There is no OECD TG available to measure photocatalytic activity. One ISO standard (ISO, 2007b) is available for fine ceramics. ISO is developing a document on “Photocatalytic activity assay for nanoparticles in aqueous suspension” (ISO, 2016c).

Within the Testing Programme the following methods were applied, see also Table 3:

#### 6.3.1 NADH monitoring

Monitoring photo-induced oxidation of Nicotinamide Adenine Dinucleotide (NADH) was applied to TiO_2_.

The method is being taken forward by ISO and an International Standard is under development whose aim is to measure the photocatalytic activity (PCA) of TiO_2_ nanoparticles in aqueous suspension by using the photo-induced oxidation of NADH, which can be observed through the loss of NADH fluorescence in the presence of photo-excited NMs. PCA of TiO_2_ nanoparticle suspensions at various conditions can be measured simultaneously allowing a fast and accurate photo-oxidation rate measurement.

#### 6.3.2 Rhodamine-B dye degradation

Rhodamine-B dye degradation was applied to CeO_2_ and ZnO. Rhodamine-B dye degradation in the presence of nanomaterials under simulated sunlight using UV-Vis spectroscopy was found to be suitable and sufficient for measuring photocatalytic activity of nanomaterials in colourless and light-coloured suspensions, presumably with some loss of sensitivity for turbid suspensions. However, this technique is unsuitable for nanomaterials that form coloured suspensions, as this interferes with the UV-Vis spectroscopy. In such cases centrifugal ultrafiltration is typically used to remove nanoparticles and allow for analysis of the dye degradation.

#### 6.3.3 DPPH degradation

Similar to Rhodamine-B dye degradation, DPPH (2,2-diphenyl-1-picrylhydrazyl) degradation measurement under UV light is suitable for nanomaterials in lightly coloured suspensions, but not for nanomaterials in coloured suspensions. DPPH degradation was applied to CeO_2_ and ZnO.

#### 6.3.4 Electron paramagnetic resonance (EPR)

The generation (or not) of hydroxyl radicals by the presence of TiO_2_ (NM-105 [Anatase ca. 84% and rutile ca. 16%], NM-103 and NM-105 [both rutile]) and irradiation with UV light was successfully measured with EPR. There was insufficient information available in the dossier to evaluate its suitability to other nanomaterials.

#### 6.3.5 Orange II degradation detected by UV-Vis spectroscopy

The degradation of Orange II under UV exposure in the presence of TiO_2_ (NM-105 [Anatase ca. 84% and rutile ca. 16%]) was detected by UV-Vis spectroscopy. There was insufficient information available in the dossier to evaluate its suitability to other nanomaterials.

#### 6.3.6 Degradation of formaldehyde

The degradation of formaldehyde in the presence of TiO_2_ (NM-105 [Anatase ca. 84% and rutile ca. 16%]) was investigated in the Testing Programme, but there was insufficient information available in the dossier to evaluate its suitability to other nanomaterials.

#### 6.3.7 Degradation of acetaldehyde under UV-light and CO_2_

ISO 22197:2 (ISO, 2011) was evaluated for SWCNTs, MWCNTs and C_60_. This method determines degradation of acetaldehyde in the presence of nanomaterials, measured under UV light and CO_2_, using gas chromatography. Apart from carbon nanomaterials, the experts indicated that the method would be suitable for (metal-)oxide-like forms of nanomaterials (OECD, 2016b), noting that the method was found to be insufficient for quantitative measurements, which may be improved by optimisation of the sample preparation.

OECD (OECD, 2016b) concluded that all the methods for measuring photocatalytic activity mentioned above are to be considered screening methods for photo-active materials, and are not suitable for quantitative/normative measurements. An option could be to identify/compare/quantify radical type generated by photoactive materials. Furthermore, it should be noted that, in general, photocatalytic activity is difficult to assess, and many methods can only determine an “apparent photocatalytic activity” as measured values refer to the total sample composition including impurities with known high photocatalytic activity (e.g. CNTs with catalyst impurities).

#### 6.3.8 Methods not used in the Testing Programme

Other possible chemical detection methods exist such as the Griess reaction (for detecting the presence of nitrite ion in solution) (Griess, 1879), and one should be aware of interference and possible errors (e.g. Kampfer et al., 2017).

## 7 Discussion

The Testing Programme has helped in answering some questions regarding the role of physico-chemical parameters for regulatory purposes (e.g. the limits for using of the octanol-water partitioning coefficient in the assessment of nanomaterials) and the availability of Test Guidelines or standardised methods. Additional methods not applied in the Testing Programme provide further insights towards new and/or better methods that may be needed for regulatory purposes, and may thus be used for updating or developing new Test Guidelines or other standardised methods.

There are already regulatory requirements to generate physico-chemical characterisation data on nanomaterials, as e.g. EU regulations may require information on nanomaterials (e.g. EC, 2012, EC, 2009), or the US EPA's rule for reporting and recordkeeping requirements for chemical substances when manufactured or processed as nanoscale materials. Nanomaterials therefore need to be detected, identified, sometimes quantified, and properties relevant for a safety assessment need to be known.

Based on this information and the analysis, the following observations can be given.

### 7.1 Regulatory relevance of physico-chemical parameters

All physico-chemical endpoints proposed by the WPMN and included in the Testing Programme are of regulatory relevance for nanomaterials, except for the pour density, which was only sparsely tested and is not a much-used parameter in the safety assessment. For the water-octanol partitioning coefficient in was noted that although required for chemicals, it is meaningless for particulate, insoluble nanomaterials (for soluble particles usually the same approach applies as for conventional chemicals). Whereas it would be relevant to be able to measure relative hydrophobicity/hydrophilicity, no generally accepted methods were identified for nanomaterials.

Size (and size distribution) stood out as the common characteristic of all nanomaterials and as such is decisive information for classifying a material as a nanomaterial.

Surface functionalization of nanoparticles usually implies that the functionalised surface has different properties from the core of the particle, thus influencing the behaviour of the particle. As such it is an important particle descriptor for nanomaterials (e.g. ECHA, 2017b). The Testing Programme did not have a strong focus on surface functionalization, and given the importance of this aspect, more work would be needed to enhance the understanding. In some applications of nanomaterials, surface functionalization is highly interesting e.g. in nanomedicine, where different layers allow to steer the transport of nanomedicines in the human body.

Though not listed as a parameter in the originally proposed set of additional endpoints for nanomaterials, shape is an important particle descriptor and should also be included in the data relevant for nanomaterials (e.g. ECHA, 2017b).

The other physico-chemical endpoints are relevant to understand nanomaterials’ interaction with the surroundings, which is important for hazard and safety assessment. Depending on the specific nanomaterial and type of assessment, not all parameters would always be required. Nevertheless for (environmental) fate and toxicokinetics dissolution and dispersibility in relevant media appear of particular relevance as these parameters inform on sedimentation and residence time in these media. For reactivity the redox potential and radical formation potential appear essential. A careful evaluation of the information needed for a specific purpose, possible methods, and available equipment should be done before embarking on physico-chemical characterisation. One recurrent conclusion within the WPMN is that for any kind of testing of nanomaterials, sample preparation is a key step, which should be carefully planned and documented in detail, and the WPMN published guidance for sample preparation and dosimetry (OECD, 2012).

Apart from (regulatory) relevance, reliability and validation of test methods are extremely important aspects in regulatory testing. In this context relevance can be defined as a description of a relationship of the test to the effect (or property) of interest and whether it is meaningful and useful for a particular purpose. It is the extent to which the test correctly measures or predicts the (biological) effect or property of interest. Relevance incorporates consideration of the accuracy (concordance) of a test method. Regulatory need, usefulness and limitations of the test method are aspects of its relevance. Reliability is a measure of the extent that a test method can be performed reproducibly within and between laboratories over time, when performed using the same protocol. It is assessed by calculating intra- and inter-laboratory reproducibility and intra-laboratory repeatability. Validation is the process by which the reliability and relevance of a particular approach, method, process or assessment is established for a defined purpose (OECD, 2005, EURLAB, 1996, ASTM, 2009). Based on these definitions, most of the methods described are relevant and reliable, but not validated. It is evident from the Testing Programme that many methods need to be adapted to be suitable for nanomaterials.

### 7.2 Availability of methods to determine physico-chemical properties of nanomaterials

For most of the endpoints there are several methods available to measure them in a quantitative way (Table 1 to Table 3). This is specifically true for the inorganic carbon and metal oxide nanomaterials investigated in the Testing Programme. For other material types there may be gaps, but the Testing Programme addressed a priority list of 11 Manufactured Nanomaterials for testing based on materials which are in, or close to commercial use, and these were mostly inorganic carbon, metal and metal oxide nanomaterials and only included nanoclays and dendrimers as other materials, and thus a detailed analysis of available methods for other material types was not performed.

It should be noted that many of the techniques and methods employed are not specific to nanomaterials; they are generally applicable to chemicals including nanomaterials.

The Testing Programme sparked method development and e.g. a new technique for determining volume specific surface area, Transmission Electron Tomography, based on TEM, was developed and investigated in the Testing Programme for SiO_2_ (van Doren et al., 2011). Additionally, a new semi-automatic method for measuring particle size distribution with TEM was proposed (de Temmerman et al., 2012, de Temmerman et al., 2014). Another example is dustiness testing where the European Committee for Standardisation (CEN) is initiating a nanospecific version of EN 15051 (British Standards, 2013b).

Details of the applicability of the different methods are given in the text, indicating that most of the available methods have limits regarding the materials to which they can be applied, or test conditions, etc. More work on experimental methods is needed, in particular to determine the surface chemistry (the chemical nature of the particle surface), dissolution (rate) and aggregation state in relevant media. Some methods are standardised already, sometimes for testing specific materials (e.g. ISO, 2016b). The WPMN has initiated an activity to adapt TG 110 (Particle Size Distribution/Fibre Length and Diameter Distributions) to make it applicable to nanomaterials. ISO and CEN have also initiated a number of projects for providing additional standardised methods for nanomaterials characterisation (e.g. www.iso.org/committee/381983.html).

### 7.3 Standardisation of methods to determine physico-chemical properties of nanomaterials

The data for a set of physico-chemical properties of manufactured nanomaterials, which were generated within the Testing Programme by applying various methods (OECD, 2010, OECD, 2016e) are regarded as particularly important for the insights they provide in characterisation of manufactured nanomaterials. When checking the OECD TGs against the proposed physico-chemical endpoints relevant for nanomaterials, it is evident that only few of the current OECD TGs are relevant for measuring these properties. Of the relevant TGs few are applicable and some are only applicable with limitations (OECD, 2009). Therefore, an assessment of the regulatory need for these endpoints is relevant, before considering development of new OECD TGs or Guidance Documents or other harmonised and standardised methods.

One feature common for most of the methods described here is that their outcomes can be strongly influenced by both by the specimen preparation and the examined sample itself. For example for tests requiring dispersion in liquid medium, the dispersion procedure and dispersion stability are major factors influencing the outcomes. The stability of NM dispersions is in particular affected by surface charge, but also by particle size and chemistry at the particle surface, which all may change the particle size distribution by aggregation, agglomeration and/or sedimentation. Sedimentation due to irreversible agglomeration of smaller particles is an indicator of true instability, but often sedimentation occurs because the primary particles or primary agglomerates are sufficiently large and/or heavy that gravity can overcome the back-diffusion due to Brownian motion. Consequently, it is uncertain to which extent the test results are relevant for the properties of the materials in isolation and/or in the presence of biological media. Although similar statements could be made for chemicals in general, the WPMN recognised the particular importance of these issues for testing nanomaterials and subsequent modelling of (environmental) fate and toxicokinetics and toxicity.

Therefore the WPMN produced, in parallel with its Testing Programme, the “Guidance on Sample Preparation and Dosimetry for the Safety testing of Manufactured Nanomaterials” (OECD, 2012). It is regarded as one of the major outcomes of the Testing Programme. The need to include standardised sample preparation procedures in any new or revised TG that addresses manufactured nanomaterials seems evident, and should not compromise the inherent flexibility of a guideline and the possible need to adapt them depending on the tested material. Recent publications (e.g. Hartmann et al., 2015) and EU projects (e.g. NANoREG, NanoDefine) address this need.

In addition to methods under development in the OECD TGP, also the standard developing organisations are standardising methods and techniques for detection of nanomaterials in (complex) matrices, e.g. ISO (e.g. ISO, 2016b, ISO, 2016e, ISO, 2017a), ASTM International (formerly American Society for Testing and Materials), CEN (e.g. CEN, 2016), and Japanese Industrial Standards. In addition, both CEN and ISO are proposing “measurement matrix” reports that provide an overview of methods and measurands applicable to physico-chemical characterisation of nanomaterials and (additional) methods are contributed by research projects, e.g. NANoREG and NanoDefine that may provide a basis for standardisation.

Some of the physico-chemical properties of nanomaterials do not fall within the area typically covered by the OECD TGP; some of the TGs that could be applicable were evaluated, reaching the following conclusions: TG 105 (addressing water solubility) was evaluated to not be applicable for nanomaterials (OECD, 2009, OECD, 2014b) in its current procedure. For nanomaterials that disperse into small primary nanoparticles the elution method in TG 105 needs to be adapted with appropriate particle detection methods. It has been proposed as a candidate for updating (OECD, 2016b) and is awaiting a volunteer lead. Activities for revision of TG 110 (on Particle Size Distribution/Fibre Length and Diameter Distribution) to address specifically nanomaterials have been initiated, the outcome could be either a revised TG or a complementing GD.

OECD TGs for determination of characteristics such as surface area, zeta-potential, redox potential, surface reactivity etc. would fit very well into an extended set of TGs for physico-chemical characterisation that support the hazard and risk assessment of manufactured nanomaterials. As for solubility for nanomaterials it is important to distinguish between solubility and dispersibility, OECD initiated several interlinked projects that will result in several new TGs and GDs for characterising nanomaterials in the aquatic environment:

• A TG on Dispersion Stability of Nanomaterials in Simulated Environmental Media which was finalised by the OECD Test Guidelines Programme in April 2017 (publication on the OECD website is expected for September 2017).
• A new TG on Dissolution Rate of Nanomaterials in the Aquatic Environment
• A new TG for Nanomaterial Removal from Wastewater
• A new GD on Agglomeration and Dissolution Behaviour of Nanomaterials in Aquatic Media

Development of additional TGs and GDs are under consideration by the OECD Member Countries.

These new documents fit to the current scope of the OECD TG series 300 that relate to environmental fate and behaviour of chemicals.

### 7.4 Data, measurements and data quality

When performing measurements, it is not always possible to directly measure the property of interest (the quantity intended to be measured), which for nanomaterials could be size, shape, chemical composition, surface charge, etc. Often the instruments do not directly measure the relevant property and a conversion of the instrument's measurand is needed to obtain information on the property of interest. An example is DLS which directly measures the intensity of scattered light that is then converted to desired information, i.e. particle size. In the conversion it is assumed that the measured particles are spherical, which may result in errors in particle sizes determined, when the particles in question have other shapes. Therefore, the converted results need to be carefully interpreted, taking into account uncertainties, limits and bias originating both from the indirect measurement and the conversion (Babick et al., 2016).

The data provided in the dossiers were not always sufficiently documented to allow evaluation of the (more general) suitability of the method. Information on chemical composition and especially the presence of impurities including residual materials or by-products, etc. was not always provided. This information gap may affect the interpretation of results from the tests for physico-chemical properties as well as the tests pertaining to toxicity, ecotoxicity and fate where the presence of impurities could have an impact. It appears necessary to agree on the details of the information to be reported in future studies on nanomaterials, and it seems relevant to establish mechanisms with the goal to generate data for which sufficient detail is provided according to previous agreements. An important step towards agreement on reporting information is the development of OECD Harmonised Templates (OHT) for some of the endpoints (OECD, 2016f). Additional data logging templates for environmental, health and safety assessment of nanomaterials have been proposed in the EU FP7 project NANoREG (Totaro et al., 2017) and are implemented in the eNanoMapper database available at https://data.enanomapper.net/.

## 8 Concluding remarks

The evaluation of available methods for the characterisation of physico-chemical parameters shows that there is quite a variety of methods available to characterise materials according to properties thought to be relevant to assess their safety. However, there is a need for further standardisation of methods and even development of new methods for some parameters.

For nanomaterials a particular characterisation challenge is the (potential) need to characterise them at several stages, e.g. as pristine material (for regulatory identification), as prepared for testing (for identifying e.g. interactions with the test media), and in situ while testing to identify any adverse effects. Furthermore, for (eco)toxicological testing, for nanomaterials the usefulness of the classical regulatory dose metrics (mg/kg or mg/L) is questioned and uncertainties about the best dose metric still exist (SCENIHR, 2009 [p.13]), and e.g. surface area has been suggested (Schmid and Stoeger, 2016). The insights obtained through the WPMN testing were consolidated in “Guidance on Sample Preparation and Dosimetry” (OECD, 2012).

Notably, the WPMN has initiated work for developing guidance on Principles for Measurement and Reporting for Nanomaterials and a Physico-chemical Decision Framework to Inform Decisions for Risk Assessment. Furthermore, the publication of standardised sample preparation procedures, frameworks and toolboxes for nanomaterial's assessment that is (being) developed (e.g. in various EU funded projects), is already available (Jantunen et al., 2017) or is imminent.

When available, such standardised methods, procedures and guidance, will certainly support the application of the agreement on Mutual Acceptance of Data (MAD) and improve our capacity to perform meaningful assessment of nanomaterials.

The urgency of solving these issues is evident also from a regulatory point of view, e.g. in the light of REACH information requirements.

## Disclaimer

Any opinions expressed in this publication are those of the authors only. For Kirsten Rasmussen, Hubert Rauscher, Agnieszka Mech, Douglas Gilliland and Juan Riego Sintes, this paper does not represent an official position of the European Commission.

## Funding

This research did not receive any specific grant from funding agencies in the public, commercial, or not-for-profit sectors.
