# Supplementary material for: Physico-chemical properties of manufactured nanomaterials - Characterisation and relevant methods. An outlook based on the OECD Testing Programme
Source: Regul Toxicol Pharmacol. 2018 Feb;92:8–28. doi: 10.1016/j.yrtph.2017.10.019 (PMC5817049; doi:10.1016/j.yrtph.2017.10.019)
Supplement: Supplementary [file mmc1.docx]

**Description of Methods other than OECD Test Guidelines applied in the OECD WPMN testing (in alphabetical order)**

Note: the methods described here are the ones used in the WPMN testing programme.

Other methods mentioned in the main article, but not used in the testing programme, have literature references associated in the main article.

| Atomic force microscopy, AFM | See Scanning probe microscopy (SPM). |
| --- | --- |
| Barrett-Joyner-Halenda method, BJH | The method of Barrett, Joyner, and Halenda is a procedure for calculating pore size distributions from experimental isotherms using the Kelvin model of pore filling. It applies only to the mesopore and small macropore size range. The Kelvin equation describes the change in vapour pressure due to a curved liquid–vapour interface, e.g. the surface of a droplet. The vapour pressure at a convex curved surface is higher than that at a flat surface. The Kelvin equation relates to thermodynamic principles and not to special properties of materials. It is also used for determination of pore size distribution of a porous medium using adsorption porosimetry. |
| Benzoic acid in PBS | The technique of using Benzoic acid in a phosphate buffered hydrous solution (PBS) comprises the formation of 4-hydroxy-benzoic acid in PBS, which is detected by HPLC-UV, under the assumption that hydroxyl radicals are the principal free radicals produced from the particle surface, which may not always be a valid assumption. The test was found unsuitable for the evaluated nanomaterial, synthetic amorphous silicon dioxide. |
| Brunauer-Emmett-Teller method, BET | The method of Brunauer, Emmet, and Teller is used to determine surface area using a model of adsorption which incorporates multilayer coverage. BET is based on the measurement of adsorption isotherms of an inert gas (typically N_2_) on a dry solid material, and then calculating, via the BET equation, the surface area of the solid. The BET equation does not provide a pore size or a pore size distribution. An average pore diameter is calculated based on the assumption that pores are uniform and cylindrical. |
| Centrifugal liquid sedimentation, CLS | CLS is forced sedimentation for measuring particle sizes. The technique does not distinguish individual particles from aggregates or agglomerates. The centrifugation force separates the particles according to their size and density, allowing to investigate the size distribution. CLS may be prone to errors arising from incorrect density values (of the sample and the medium) and where optical detection is used to quantify mass the optical properties are critical. |
| Continuous drop | The continuous drop method is a test method for dustiness according to the CEN standard EN 15051 “Workplace atmospheres—Measurement of the dustiness of bulk materials—Requirements and reference test methods”; the apparatus used is described as reference test method B in EN 15051 (see Bach and Schmidt, 2008, [10.1093/annhyg/men062](https://doi.org/10.1093/annhyg/men062))  A dustiness tester consists of two parts, a dust generator and a dust sampler. The design of the dust generator in the drop method is based on the principles of gravity. In the continuous drop method the bulk material is transported from a storage tank via the metering device through the drop pipe into the flow of the back flow pipe. The drop mass flow can be varied by changing the speed of the metering device and is to be set by weighing to a value between 6 and 10 g min^−1^ ([EN 15051, 2013](http://shop.bsigroup.com/ProductDetail/?pid=000000000030252269)). The constant back flow has a velocity of 0.05 m s^−1^ and is drawn in via drill holes in the detachable collector tank. The inhalable and respirable aerosol fractions are separated simultaneously by partial flow pumps to adequate filters mounted in the sampling heads. |
| Differential scanning calorimetry, DSC / Thermogravimetric Analysis, TGA | Differential Scanning Calorimetry (DSC) and Thermogravimetric Analysis (TGA) are methods which, respectively, measure specific heat capacity or weight of a test sample when undergoing controlled heating. In DSC the difference in the amount of heat required to increase the temperature of a sample and a reference is measured as a function of temperature, and DSC is used to identify phase transitions. In TGA changes in mass of materials are measured as a function of increasing temperature (with constant heating rate), or as a function of time (with constant temperature and/or constant mass loss). TGA can provide information about physical phenomena, such as second-order phase transitions, including vaporization, sublimation, absorption, adsorption, and desorption. |
| DPPH degradation | DPPH (2,2-diphenyl-1-picrylhydrazyl) is characterized as being a stable free radical as a result of the delocalisation of the spare electron over the molecule as a whole, meaning that the molecules, in contrast to most other free radicals, do not dimerise. The delocalisation also gives rise to the deep violet colour, with an absorption in ethanol solution at around 520 nm. On mixing DPPH solution with a substance that can donate a hydrogen atom, it gives rise to the reduced form with the loss of violet colour degradation measurement under UV light. |
| Dynamic light scattering, DLS | DLS measures the equivalent hydrodynamic diameter (i.e. the diameter of a spherical particle moving as an individual unit in a liquid surrounded by a solvation layer) by measuring fluctuations of light scattered from the sample and gives a scattered light intensity based size distribution. A mathematical transformation of the intensity-based distribution is applied to calculate the particle number size distribution (i.e. number of particles and agglomerates/aggregates) and particle volume size distribution (i.e. volume of particles and agglomerates/aggregates). In most of the nanoscale size range the scattered light intensity is proportional to the sixth power of the size of the scatterers (the particles), and therefore the signal of small particles can easily be outshined by that of large particles. If this is the case, the transformation from an intensity based distribution to a number or volume based distribution does not give true values. However, if the size distribution is narrow or quasi-monodisperse, the light scattering techniques can render reasonable results. The instrument’s software evaluates the scattered intensity usually assuming spherical particles. This means that also for non-spherical particles a spherical equivalent hydrodynamic diameter is reported, which must be kept in mind when drawing conclusions from such results. Additionally, this technique requires information about viscosity of the medium and refractive index (for the conversion from intensity to mass) as well as information on the optical absorption coefficient (imaginary part of refractive index) of the test material, which for some samples can be difficult to obtain. |
| Electrophoretic light scattering for Zeta potential measurement | Particles in suspension will often have an electrical double layer around them which consists of two parts, the inner region called the stern layer, where the ions are strongly bound, and the outer, called the diffuse region, where they are less firmly attached. When a particle moves the ions within a boundary in the diffuse layer will move with it, but any ions beyond it do not travel with the particle. This is known as the slipping plane, the zeta potential is the potential that exists at this boundary.  Since zeta-potential is a measure of the magnitude of the electrostatic or charge repulsion or attraction between particles in a liquid suspension it is one of the fundamental parameters known to affect dispersion stability. Its measurement provides a detailed insight into the causes of dispersion, aggregation or flocculation, and can be applied to improve the formulation of dispersions, emulsions and suspensions.  To determine the zeta-potential of a nanoparticle dispersion it is necessary to measure the related parameter, electrophoretic mobility, using the technique of Electrophoretic light scattering (ELS).  This is done by introducing the dispersion into a cell containing two electrodes and applying an electrical field to the electrodes. Under the influence of the electrical field, charged particles or molecules will migrate towards the oppositely charged electrode with a velocity which is related to the electrophoretic mobility. If the field strength is known and the speed of movement measured using laser light scattering methods (e.g. Laser Doppler velocimetry) then the electrophoretic mobility can be determined and then used to calculate the zeta-potential using a model such as that based on Smoluchowski theory. |
| Electron Microscopy, EM | **Electron Microscopy**  Electron microscopy (EM) refers to a number of specific techniques all of which use an electron beam to illuminate a specimen and produce a magnified image. The use of an electron beam rather than light permits the visualisation of objects and structures with dimensions much below that which is physically possible by any optical microscopy technique. Transmission electron microscopy (TEM) and scanning electron microscopy (SEM) are the most common methods in use, the first being capable of atomic resolution, the latter having a resolution limit of around 5nm. In general, both techniques give a two-dimensional projection of the three-dimensional object(s) of interest. EM based methods have advantages over most other particle sizing and detection methods, in that they can provide information about the size and shape of individual particles and are able (sometimes with some limitations) to distinguish and characterise primary (constituent) particles within larger aggregates/agglomerates (especially high resolution TEM). The major disadvantages of EM methods are the need to analyse samples under high vacuum, the high purchase and running costs, and the relatively slow throughput.  **SEM**  For SEM analysis, the sample is mounted in a high vacuum chamber and the surface scanned line by line with a finely focussed electron beam. As the beam scans over the sample, electrons are emitted locally from the surface and their instantaneous intensity measured by specific electron detectors. The electrons leaving the sample surface are of two types: firstly there are electrons originating from the electron beam which are scattered from the sample surface (backscattering electrons – BE) and secondly there are electrons originating from the sample itself (secondary electrons – SE) which are ejected from the surface following inelastic scattering interactions with the beam electrons.  As the beam is scanned over the sample in a raster pattern the intensity of each type of emitted electrons is measured by detectors and the intensity combined with the beam's position to produce an image. As BE and SE intensity varies with local topography and elemental composition, the resulting 2-D image provides information on the surface topography and composition.  Depending on the sample type SEM can achieve a spatial resolution of around 5 nm or better. SEM instruments are often integrated with EDAX X-ray detectors (see below) which allow elemental analysis by collecting the X-rays emitted by the sample when interacting with the electron beam.  **TEM**  In TEM imaging, a small, thin, coherent beam of electrons is transmitted through an ultrathin specimen where it interacts with the specimen. As the beam passes through the thin sample, local differences in thickness and composition produce variation in the intensity of the transmitted electron beam. The resulting contrast represents an image of the sample which can be magnified and focused by an objective lens onto an imaging screen (a fluorescent screen in most TEMs) for direct observation by the operator or alternatively onto a imaging sensor such as a charge coupled device (CCD) camera.  For TEM analysis to be possible the samples to be analysed must be sufficiently thin as to make them be partially transparent to the electron beams. Achieving this pre-requisite requires special sample preparation   - Fine particles may be directly deposited from dispersion onto a thin support film held on a metallic grid. - Biological samples may be embedded in a resin block which is then cut into thin slices using an ultramicrotome. The resulting thin films may then be directly mounted on a metallic support grid. - Bulk samples that cannot be cut by an ultramicrotome may be thinned locally using techniques such as mechanical milling, or alternatively etching by chemical or ion beam methods. - For purely topographical analysis of the surface of a bulk sample the technique of replication may be adopted. In this procedure the surface of the sample is conformably coated with a layer of a polymer such as cellulose acetate. The coating can then be stripped from the sample surface to expose the polymer surface which retains a 3-D negative representation of the original sample surface. This surface is then coated with a thin metallic layer after which the polymer can be dissolved to leave a thin metallic layer which is a replica of the original sample surface.   Depending on the sample types TEM analysis can achieve a spatial resolution of around 1 nm for standard instruments while more complex, high resolution instruments may reach atomic scale resolution.  TEM instruments are often integrated with EDAX X-ray detectors (see below) which allows elemental analysis by collecting the X-rays emitted by the sample when interacting with the electron beam. |
| Electron paramagnetic resonance (EPR) / Electron spin resonance (ESR) | Via EPR / ESR materials with unpaired electrons can be studied. The technique works in the following way: a nitrone or nitroso compound (a spin trap) reacts with a target free radical which forms a stable and distinguishable free radical (spin adduct) that can be detected directly by ESR spectroscopy. The ESR spectra of these spin adducts are unique and provide a “fingerprint” for the presence of the ROS. |
| Energy dispersive X-ray analysis EDX (EDS, EDXS or XEDS), sometimes called energy dispersive X-ray analysis (EDXA) or energy dispersive X-ray microanalysis (EDXMA) | Energy dispersive X-ray analysis is a generic analytical method for measuring the intensity and wavelength spectra of X-rays. The method is typically based on the use of lithium doped semiconductor detectors (Si(Li) or Ge(Li)) or microfabricated chips (e.g. Silicon Drift) which convert X-ray photons to an electrical signal which is used to produce an X-ray energy spectrum.  EDAX detectors are commonly used in elemental analysis instruments where the samples to be analysed are made to interact with a source of X-ray excitation which stimulates the emission of X-rays from the sample. Since each element in the periodic table has a unique electronic structure the X-rays energy spectrum emitted by a sample can be used to firstly identify the individual elements present in the sample and then calculate the relative mass concentration of each.  EDAX detectors are often used in electron microscopy (SEM and TEM) instruments where the imaging electron beam is also an effective means of generating element specific X-rays from the samples. Alternatively, EDAX is also commonly used in X-ray florescence spectroscopy for elemental analysis. In this case the characteristic element specific X-rays emitted by a sample are produced by illuminating the sample with a high energy X-ray source or gamma-ray source. |
| HCHO degradation | Formaldehyde is known to photodegrade in the presence of TiO_2_. |
| ICP (ICP-MS, and ICP-OES) | ICP-OES (also known as ICP-AES) and ICP-MS are techniques used for trace and ultra-trace elemental analysis. Each technique combines a spectrometric detection and quantification instrument with a high temperature Inductively Coupled Plasma (ICP) torch for sample vaporization and excitation prior to analysis.  The component which is common to both methods, the Inductively Coupled Plasma torch, uses an intense electromagnetic field to generate a high energy plasma in a flow of argon gas. This resulting high temperature flame (6000-10000K), which is composed of mainly argon atoms with a small proportion of argon ions and free electrons, provides a highly energetic environment capable of atomizing and ionizing any sample introduced into it. In most cases the samples to be analysed are in the form of a liquid solution which is pumped into an analytical nebulizer to form a fine mist which is then introduced directly into the high temperature plasma flame. Once inside the plasma flame the fine mist is vaporized and the dissolved material atomized and ionized.  Alternatively, some instruments are equipped with a laser ablation system which provides a means to introduce material into the plasma flame directly from solid samples. In this method, a pulsed UV laser is focused on the sample and creates a plume of ablated material which can be swept into the plasma without the need to dissolve the sample.  In the case of the ICP-OES technique, the analyte atoms in the plasma flame absorb energy to become excited/ionized before decaying to lower energy states through optical emission at wavelengths characteristic of the elements present. This optical emission is then analysed using an optical spectrometer to firstly identify the elements present and then after suitable calibration used to quantify the amount of each element. In the case of the ICP-MS technique the plasma flame serves to produce ionized atoms of the analyte which, being electrically charged, can be extracted from the flame and introduced into a mass spectrometer for identification and quantification. |
| ISO 22197-1 / JIS R 170-1 | ISO 22197-1:2007 specifies a test method for the determination of the air-purification performance of materials that contain a photocatalyst or have photocatalytic films on the surface. These films are usually made from semiconducting metal oxides, such as titanium dioxide or other ceramic materials, by continuous exposure of a test piece to the model air pollutant under illumination with ultraviolet light. ISO 22197-1:2007 is intended for use with different kinds of materials, such as construction materials in flat sheet, board or plate shape, that are the basic forms of materials for various applications. It also applies to materials in honeycomb form, and to plastic or paper materials if they contain ceramic microcrystals and composites. It does not apply to powder or granular photocatalytic materials.  This test method is usually applicable to photocatalytic materials produced for air purification. This method is not suitable for the determination of other performance attributes of photocatalytic materials, i.e., decomposition of water contaminants, self-cleaning, antifogging and antibacterial actions. It concerns the removal of nitric oxide. |
| KI-test | See potassium iodide test. |
| Laser diffraction | Laser diffraction, also known as static light scattering, is a commonly used method for measuring the size and size distribution of particulate materials. Laser diffraction measures particle size distributions by measuring the angular variation of the intensity of light scattered by a laser beam as it passes through a dispersed sample of particulates. The samples analysed are commonly prepared as dispersions of solids in a compatible liquid, but suitably equipped instruments can also analyse dry particulates when dispersed in a flow of gas. Large particles scatter light at small angles relative to the laser beam and small particles scatter light at large angles. The angular scattering intensity data is then analysed using light scattering theory (e.g. Lorenz-Mie and Fraunhofer diffraction) to calculate the size of the particles responsible for creating the scattering pattern. The particle size is reported as a volume equivalent sphere diameter and is generally limited by the lower size which can be detected, which commonly is around 50 nm. The effective limit depends on a number of factors such as wavelength of the laser used and the optical properties of the materials and its dispersion media, but in any case is seldom much lower than 10 nm. |
| Mass spectrometry, MS | In mass spectrometry (MS) the sample is ionized, and the ions sorted based on their mass to charge ratio, and a mass spectrum measures the mass/charge combinations generated within a sample.  See also ICP-MS. |
| Mercury Intrusion Porosimetry | Mercury Intrusion Porosimetry is based on the non-wetting mercury properties which, combined with the high surface tension of mercury, at high pressure allows intrusion of mercury into the pores of a material. The external pressure needed to force the mercury into the pores against the opposing force surface tension of the liquid is used to determine the pore size. It has been standardised by ISO ([ISO 15901-1:2016](https://www.iso.org/standard/56005.html)) and an earlier version of the standard was applied in the Testing Programme. |
| Nicotinamide Adenine Dinucleotide (NADH) monitoring | NADH monitoring aims to measure photocatalytic activity (PCA) of nanoparticles in aqueous suspension by using the photo-induced oxidation, e.g. via an UV light source, of NADH, which can be observed through the loss of NADH fluorescence in the presence of photo-excited nanoparticles (NPs). The NADH fluorescence intensity-decrease before and after successive irradiations of UV light is observed, which allows to determine the fraction of the oxidized NADH due to the photocatalytic action of NPs. The photo-oxidation rate of NADH is determined at several NP concentrations, due to the self-masking effects by NP, in a dilution series. The NP concentration range shows linear dependence of the photo-oxidation rate of NADH vs. NP concentration, where the slope provides the NADH photo-oxidation rate per unit measure of NP in aqueous suspension. |
| Nuclear magnetic resonance, NMR / Diffusion ordered spectroscopy DOSY NMR | Nuclear Magnetic Resonance (NMR) spectroscopy is a technique that exploits magnetic properties of certain atomic nuclei (most common ^1^H, ^13^C, ^31^P) for the detailed chemical and structural characterization of molecules. It is commonly used for the chemical analysis of small organic molecules, but also used for large proteins and nucleic acids using multidimensional techniques. It is relevant to chemical and structural analysis of organic NMs, if they are not too large.  Diffusion-ordered spectroscopy (DOSY) NMR seeks to separate the NMR signals of different species according to their diffusion coefficient. DOSY is a technique for mixture analysis based on measurements of pulsed field gradient spin or stimulated echo. The measured signal intensity depends on timing, diffusion coefficient, and gradient amplitude. Performing measurements with a range of gradient strengths means that the diffusion coefficients for different signals can be determined. Given that the diffusion coefficient is a molecule characteristic, the NMR signals from species of different sizes can be distinguished, if the signals of the organic NMs are not too broad due to the slow tumbling of the particles in solution. |
| Optical emission spectrophotometry, OES | See ICP-OES. |
| Orange II degradation | Orange II (a dye) is known to be subject to photocatalytic degradation. In the presence of a photocatalyst it will be decolourised when irradiated, which can be analysed by spectrophotometry, measuring the absorbance at λ_max_ = 483 nm. |
| Oxo-Dish, sensor disk reader, SDR | The oxo-dish method (sensor disk reader, SDR) monitors oxygen levels inside a 24-well plate during 24-hour incubation using a fluorescence method, leading to information on 24-hour hydrochemical reactivity, solubility and inferred biodurability. The SDR system enables simultaneous measurement in 24 wells at one second resolution and therefore ability to establish a variety of data as function of dose and time. The test conditions for the SDR system are maintained by a cell-incubator and consequently directly corresponds to the conditions of a given in vitro exposure event. However, one drawback is that the measurable pH-range is limited to pH 5 to 9. The range in O_2_ concentrations is wide and varies from 0 to 250% O_2_ saturation (0 to 707.6 µmol/L). Due to the principle link between electron activity and oxygen fugacity (e.g. Nordstöm and Munoz, 1994), the variation in O_2_ may correspond to values obtained by direct redox potential measurement.  Data on the hydrochemical reactivity and biodurability of NMs may be important to better understand the biochemical reactivity of nanoparticles and dissolution in contact with specific biofluids. When particles come in contact with biofluids, reactions may take place that cause e.g. changes in pH, adsorption of ions or biomolecules, dissolution, and electron loss or gain, which can result in formation of reactive oxygen species (ROS). ROS are often considered as being one of the most important parameters of hydrochemical reactivity.  As an additional output from the SDR studies, the measured amount of soluble NM (concentrations of dissolved elements) after the 24-hour incubation in each incubation medium may be reported. For this, liquid samples should be carefully extracted, filtered and centrifuged to remove dispersed NM in the liquid sample, and quantified, e.g. by ICP-OES and ICP-MS. The concentrations of dissolved elements give indication on the durable fraction (total minus the dissolved amount) in each medium; for quantitative results a high-precision analysis on the starting materials is needed. |
| Polarised Light Optical Microscopy, POM | Polarised Light Optical Microscopy (POM) covers a number of optical microscopy techniques involving polarized light. |
| Potassium iodide test, KI-test | A strategy to detect ROS formation is the use of scavenging substances that get oxidised by ROS into stable chemical compound whose concentration can be detected. One method for assessing the general oxidative activity of the NMs under irradiation is the potassium iodide (KI) test. The oxidation of iodide ions results in the production of tri-iodide ions, whose concentration can be assessed by measuring optical absorbance by UV-vis at 352 nm. For this test a solution of KI is added to a dispersion of the nanomaterial under investigation, the samples are exposed to simulated solar irradiation, which may lead to oxidation of iodide to form tri-iodide (I_3_^−^), which can be quantified by measuring UV-vis absorbance at 352 nm. In addition, positive control samples containing known concentrations of KI and anatase NM (TiO_2_), as well as negative control samples containing only KI are also prepared. All samples of a test should be prepared and analysed in the same medium. |
| Potentiometric method | The potentiometric method measures the redox potential as the potential difference across two electrodes i.e. a Pt electrode against a double junction Ag/AgCl reference electrode. |
| Raman spectroscopy | Raman spectroscopy is a technique used to observe vibrational, rotational, and other low-frequency modes in substances. It relies on inelastic scattering, or Raman scattering, of monochromatic light, usually from a laser in the visible, near infrared, or near ultraviolet range. The energy of the laser photons is shifted up or down as a result of the laser light interacting with molecular vibrations, phonons or other excitations in the system. From the shift in energy information about the vibrational modes in the system can be deducted. |
| Rhodamine B degradation | Rhodamine B ([9-(2-carboxyphenyl)-6-diethylamino-3-xanthenylidene]-diethyl­ammonium chloride) is known to be subject to photocatalytic degradation. In the presence of a photocatalyst it will be decolourised when irradiated by visible light, which can be followed by spectrophotometry, measuring the absorbance. |
| Rose bengal adsorption (RBA) | Rose bengal (4,5,6,7-tetrachloro-2',4',5',7'-tetraiodofluorescein) is a dye. Like other dyes of this class, its colour and spectroscopic characteristics mainly depend on the environment i.e. medium and presence of other compounds in the dispersion. The adsorption spectra maxima in some media are known, as is the typical changes in absorption of rose bengal caused by the presence of some materials, e.g. relevant in drug delivery.  The rose bengal partitioning method consists of addition of a rose bengal solution of fixed concentration to increasing concentrations of nanoparticle dispersions and immediate incubation of the mixture for 3 h, separation of nanoparticles by centrifugation and determination of unbound rose bengal content in the supernatant by fluorescence spectroscopy, or by UV–VIS spectroscopy; alternatively a series of rose bengal solutions with increasing concentration and fixed concentration may be tested. |
| Rotating drum | The rotating drum method for measuring dustiness is described in EN 15051. Dustiness testers consist of two parts: a dust generator and a dust sampler. The design principle for the rotating drum dust generator is mechanical dispersion. (see also small rotating drum). |
| Scanning electron microscopy, SEM | See electron microscopy (EM). |
| Scanning mobility particle spectrometry, SMPS | SMPS is a non-destructive method that requires that samples are aerosolised, and it separates the particles according to their electrical mobility, i.e. their charge. It has a relatively high resolution of approximately 2 nm and gives reliable results in the size range of 2.5 nm up to 1000 nm. The main limitation of this method is the stability of the aerosol, as SMPS results are reliable only for aerosols which are stable while scanning. |
| Scanning probe microscopy, SPM | Scanning probe microscopy (SPM) include atomic force microscopy (AFM) and scanning tunnelling microscopy (STM) which both use a scanning probe (a tip), which is moved over the surface of the sample, sensing the differences in height due to the presence of particles. In contrast to EM, scanning probe microscopy can in principle measure nanoparticles and aggregates/agglomerates in all three dimensions (maximum length, width and height), the resolution of height is better than the two lateral resolutions. |
| Scanning tunnelling microscopy, STM | See scanning probe microscopy (SPM). |
| Sensor disk reader, SDR | See oxo-dish. |
| Small Angle X-ray Scattering, SAXS | Small-angle X-ray scattering (SAXS) is a technique recording the elastic scattering of X-rays (wavelength between 0.1 to 0.2 nm) by a sample, which has inhomogeneities in the nm-range, at very low angles (typically 0.1 - 10°). SAXS is capable of delivering structural information of macromolecules between 5 and 25 nm, and of repeat distances in partially ordered systems of up to 150 nm. USAXS (ultra-small angle X-ray scattering) can resolve even larger dimensions |
| Small rotating drum, SD | The small rotating drum (SD) is a downscale of the rotating drum method described in EN 15051. See rotating drum. |
| Spectrophotometric determination of silicate according to Motomizu | Orthosilicic acid reacts with molybdate anions under strong acid conditions to form dodecamolybdatosilicateanions [SiMo_12_O_40_]^4-^. With malachite a green complex is formed. In the presence of a protective colloid (5 wt%, aqueous PVA solution) photometric determination at a wavelength of 595 nm is performed (see http://ousar.lib.okayama-u.ac.jp/files/public/1/19978/20160528004558560621/Ana_Sci_5_85.pdf). |
| Time-of-Flight, ToF | Time-of-Flight (ToF) is a separation technique that is used in connection with Mass spectroscopy (ToF-MS), and Secondary ion mass spectrometry (SIMS). |
| Time of Flight Secondary secondary ion mass spectroscopy, SIMS | Time of Flight Secondary ion mass spectroscopy (ToF-SIMS) is the dominant variant of molecular SIMS, a technique applied in analysing the composition of solid surfaces and thin films. The surface of the sample is sputtered with a focused primary ion beam and the ejected secondary ions are collected and analysed. A mass spectrometer is used to measure the mass/charge ratios of these secondary ions to determine the elemental, isotopic, or molecular composition of the surface to a depth of 1 to 2 nm. SIMS is generally considered to be a qualitative technique, due to the large variation in ionization probabilities among different materials (matrix effects). SIMS is the most sensitive surface analysis technique, with elemental detection limits ranging from parts per million to parts per billion. ToF-SIMS has the unique ability to simultaneously obtain both elemental and molecular chemical information with its spatial distribution on a sub micrometre scale (ca. 200 nm spatial resolution). Moreover, with the recent instrumental progress such as the development of cluster ion beam sources, ToF-SIMS has been successfully applied to analyse the chemical composition of both nanomaterials and materials that contain nanoparticles (see J. C. Vickerman, D. Briggs (eds.), ToF-SIMS: materials Analysis by Mass Spectrometry (2nd ed), Surface Spectra (UK), 2013; Steven G. Boxer, Annu. Rev. Biophys. 2009. 38: 53–74; S. Fearn, Materials Science and Technology 2015 VOL 31 NO 2). |
| Transmission electron microscopy, TEM | See electron microscopy (EM). |
| Turbidity | The cloudiness or haziness of a fluid caused by large numbers of individual particles that is generally invisible without magnification is called Turbidity. The technique to measure turbidity is based on determination of the concentration of suspended particles in a sample of water by measuring the incident light scattered at right angles. The scattered light is captured by a photodiode, which produces an electronic signal that is converted to a turbidity value. |
| Ultra Small Angle X-ray Scattering, USAXS | See Small Angle X-ray Scattering (SAXS). |
| Ultrasonic spectroscopy | To determine the particle size with ultrasonic techniques, the following characteristics of the sample investigated are required: the density of the solid to be determined, the concentration of the solids in the suspension, and the density of the suspending medium. The measurement range of particle size analysis is between 0.005-1000 micrometres.  The measurement principle uses the ultrasonic spectrometer DT1200 that allows the determination of the zeta potential and of the particle size distribution of particles down to the nanometre range in aqueous and non-aqueous, highly concentrated and diluted (1-50 vol%) suspensions. During the measurement, the macroscopic parameters ultrasonic attenuation, sound velocity and acoustic impedance are recorded. From these measurements the particle size can be determined by mathematical modelling. The oscillation of the charged particles in the acoustic field leads to an alternating electric field. This forms the basis for the electro-acoustic measurements and allows the determination of the zeta potential in these suspensions. The characterization of the suspension with ultrasonic spectroscopy, leads to an improved evaluation of homogeneity and agglomeration probability for the different nanoparticles in the suspension. The analysis of particle size is shown by the example of an aqueous silica Ludox suspension (standard for particle size analysis) and was calculated from the ultrasonic attenuation as a function of frequency. |
| Ultraviolet-visible spectroscopy, UV-Vis | In ultra violet-visible (UV-Vis) spectroscopy the absorption (or reflectance) of light in the ultraviolet-visible spectral region by a sample is measured by sending a beam of light in the visible and adjacent (near-UV and near-infrared) ranges through a sample in dispersion / solution and comparing to a blank (i.e. only the cuvette and the dispersion medium). The light intensity of the beam is measured before and after passing through the dispersion with the sample. The absorption may be due to plasmonic resonance effects (for Ag and Au that are plasmonic materials) or the molecular structure of the samples studied. |
| Vortex shaker method, VS | The vortex shaker method (VS) consists of a centrifuge stainless tube continuously agitated by a vortex in which the powdered test material is placed together with e.g. 100 μm diameter bronze beads used to help the de-agglomeration of powders. HEPA filtered air, controlled at 50% relative humidity, passes through the tube in order to transfer the released aerosol to the sampling and measurement section. |
| X-ray diffraction, XRD | X-ray diffraction (XRD) makes use of the fact that atoms arranged in a crystalline structure will cause an X-ray beam to diffract in well-defined directions. For powder materials, measurement of the angles and intensities of the diffracted beams yields information on the type of crystalline lattice and the associated lattice spacings. XRD patterns can be compared with diffraction databases to identify the actual material. In addition, XRD patterns of powder materials can be analysed to obtain information on crystallite size and microstrain. |
| X-ray photoelectron spectroscopy, XPS | X-ray photoelectron spectroscopy (XPS), also known as electron spectroscopy for chemical analysis (ESCA), is a powerful analytical technique that provides detailed qualitative information on the elemental composition, empirical formula, chemical state and electronic state of a material surface. Photoemission spectra are obtained by irradiating a solid surface with a beam of X-rays while simultaneously measuring the kinetic energy and number of electrons emitted from the top 1 – 10 nm of the material being analysed. Peaks appear in the spectrum at characteristic energies specific of the electronic level of the electron emitting atom. The energy position and of the intensities of the photoelectron peaks allow the identification and the quantification of all surface elements (except hydrogen) [1]. Classical XPS experiments are performed in ultra-high-vacuum, thus limiting the application of XPS to solid samples and thin films. However, the recent development of specific experimental set-up (especially at synchrotron beamlines) has permitted the exploitation of the technique also to liquid samples [2].  [1] Friedrich Reinert and Stefan Hüfner, New Journal of Physics 7 (2005) 97; S. Hüfner, Photoelectron Spectroscopy, Springer, Berlin, 1995; C.S. Fadley, Journal of Electron Spectroscopy and Related Phenomena 178–179 (2010) 2–32.  [2] M.A. Brown, et al. Physical Review X 6, 011007 (2016); J. Knudsen et al., Surface Science 646 (2016): 160–169 |
